# The expectations and acceptability of a smart nursing home model among Chinese older adults: a mixed methods study

**DOI:** 10.1186/s12912-023-01676-0

**Published:** 2024-01-13

**Authors:** Yuanyuan Zhao, Shariff-Ghazali Sazlina, Fakhrul Zaman Rokhani, Karuthan Chinna, Jing Su, Boon-How Chew

**Affiliations:** 1 The School of Smart Health and Wellness (Health Medical College), Zhejiang Dongfang Polytechnic, Wenzhou, Zhejiang Province China; 2 Department of Family Medicine, Faculty of Medicine and Health Sciences, Universiti Putra Malaysia, Serdang, Selangor 43400 Malaysia; 3 Faculty of Engineering, Universiti Putra Malaysia, Serdang, Selangor Malaysia; 4 Malaysian Research Institute on Ageing (MyAgeing TM ), Universiti Putra Malaysia, Serdang, Selangor Malaysia; 5 Faculty of Business and Management, UCSI University, Kulala Lumpur, Selangor Malaysia; 6 International School of Public Health and One Health, Hainan Medical University, Haikou, Hainan Province China; 7 Clinical Research Unit, Hospital Sultan Abdul Aziz Shah (HSAAS Teaching Hospital), Universiti Putra Malaysia, Serdang, Selangor Malaysia

**Keywords:** Smart nursing homes, Quality of care, Expectations, Acceptability, Chinese older adults

## Abstract

**Background:**

Smart nursing homes (SNHs) integrate advanced technologies, including IoT, digital health, big data, AI, and cloud computing to optimise remote clinical services, monitor abnormal events, enhance decision-making, and support daily activities for older residents, ensuring overall well-being in a safe and cost-effective environment. This study developed and validated a 24-item Expectation and Acceptability of Smart Nursing Homes Questionnaire (EASNH-Q), and examined the levels of expectations and acceptability of SNHs and associated factors among older adults in China.

**Methods:**

This was an exploratory sequential mixed methods study, where the qualitative case study was conducted in Hainan and Dalian, while the survey was conducted in Xi’an, Nanjing, Shenyang, and Xiamen. The validation of EASNH-Q also included exploratory and confirmatory factor analyses. Multinomial logistic regression analysis was used to estimate the determinants of expectations and acceptability of SNHs.

**Results:**

The newly developed EASNH-Q uses a Likert Scale ranging from 1 (strongly disagree) to 5 (strongly agree), and underwent validation and refinement from 49 items to the final 24 items. The content validity indices for relevance, comprehensibility, and comprehensiveness were all above 0.95. The expectations and acceptability of SNHs exhibited a strong correlation (*r =* 0.85, *p <* 0.01*)*, and good test-retest reliability for expectation (0.90) and acceptability (0.81). The highest tertile of expectations (X^*2*^*=*28.89, *p* < 0.001) and acceptability (X^*2*^*=*25.64, *p* < 0.001) towards SNHs were significantly associated with the willingness to relocate to such facilities. Older adults with self-efficacy in applying smart technologies (OR: 28.0) and those expressing a willingness to move to a nursing home (OR: 3.0) were more likely to have the highest tertile of expectations compared to those in the lowest tertile. Similarly, older adults with self-efficacy in applying smart technologies were more likely to be in the highest tertile of acceptability of SNHs (OR: 13.8).

**Conclusions:**

EASNH-Q demonstrated commendable validity, reliability, and stability. The majority of Chinese older adults have high expectations for and accept SNHs. Self-efficacy in applying smart technologies and willingness to relocate to a nursing home associated with high expectations and acceptability of SNHs.

**Supplementary Information:**

The online version contains supplementary material available at 10.1186/s12912-023-01676-0.

## Introduction

As the world’s second-largest economy, China is also grappling with the intricate challenge of rapid ageing [1]. According to a recent national survey (2020) based on the scale assessment for activities of daily living (ADLs) and instrumental activities of daily living (IADLs) [2], three levels were categorised based on the severity of dependency and older adults’ requirements for care. The study estimated that more than 20 million Chinese older adults were in need of minimal assistance with daily living activities, such as meal preparation and basic hygiene (level 1 dependency), 36 million needed moderate assistance with daily tasks, including cooking, shopping, and medication management (level 2 dependency), and 45 million were largely dependent on others for their daily living activities, requiring continuous supervision and assistance, such as those with severe cognitive or physical impairments (level 3 dependency), respectively. The one-child policy has directly impacted the availability of family caregivers, compounding the issue of inadequate care for Chinese older adults in their later years [3]. For the majority of older adults, dependency on assistance for daily living activities and cognitive impairments has become a significant life event, and these aspects lead to an increasing demand for nursing homes [4]. However, the quality of care provided in Chinese nursing homes is primarily influenced by policies, often falling short of meeting the demands of older adults in terms of having skilled caregivers, real-time monitoring, and continuous health assessment [5].

As sustainable strategies for promoting care for the ageing population, the use of smart technologies can address the escalating unmet healthcare needs of older adults and offset the inadequacy of medical resources to effectively improve the current healthcare system [6]. In hospital settings, smart technologies are used to enhance clinical decision-making [7], while in home-based care, they help with self-management and the remote monitoring of chronic diseases [8, 9]. In nursing home settings, technologies are predominantly implemented to provide person-centred care services and integrate medical services from remote hospitals [10]. The use of smart technologies holds the potential to support a substantial number of older adults in both home-based and nursing home-based care [11]. In 2014, the Ministry of Civil Affairs of the People’s Republic of China, the supervisory department for geriatric care, initiated the ‘Smart Elderly Internet of Things (IoT) Pilot Project’ to enhance the operation of SNHs [12]. In 2015, the Chinese government introduced the ‘Internet Plus’ plan to encourage technological innovation [13], encompassing projects related to IoT or Artificial Intelligence (AI) in safety monitoring, fall prevention, and disease detection for older adults. However, the concept of a SNH and the availability of smart technologies in nursing home settings remain ambiguous. Moreover, many older adults have a negative attitude towards smart technologies, perceiving them as challenging to use and being expensive [14]. Exploring the expectations and acceptability of SNHs within a defined service scope and associated technologies [10] among stakeholders, particularly older adults, will provide a better understanding of the future development and implementation of SNH models. Expectations, in this context, generally encompass the desires of consumers regarding what they expect a SNH to provide [15], while acceptability refers to the intention to use services when they are available and meet the criteria of target users willing to adopt SNHs [16].

Previous studies have often defined a SNH as either a smart building equipped with IoT networks [17], or the isolated application of smart technology within nursing home environment [18–21]. Specifically, a precise definition of SNHs and the comprehensive implementation of functional technologies is needed. A comprehensive scoping review has defined a SNH as characterised by the incorporating of functional information technologies, encompassing the IoT, digital health, big data, AI, cloud computing technologies, and information management system (IMS) that enable the monitoring of abnormal events, provision of remote clinical services, establishment of health information databases, enhancement of decision making processes, analysis of clinical data, and facilitation of activities of daily living for older residents [10]. It may integrate medical services from remote hospitals or healthcare experts, using telemedicine, mHealth, and other electronic clinical information, to manage complex health conditions among their residents and ensure their overall well-being within a safe and cost-effective environment [10]. Previous studies have investigated the willingness and associated factors of Chinese older adults to the conventional nursing homes [22, 23]. However, there is a lack of studies that have examined the expectations and acceptability of SNHs. It is crucial to thoroughly investigate the perspective of Chinese older adults regarding SNHs. This is necessary to ensure the successful development of innovative geriatric care models that meet the healthcare demands of China’s ageing population and are widely embraced.

### Research questions

Drawing upon the defined SNH model [10], the following research inquiries were devised: 1) What factors are important to assess the expectations and acceptability of SNHs, and their psychometric property as a tool? 2) To what extent are Chinese older adults inclined to embrace the evidence-based SNH model? 3) What are the levels of expectation and acceptability exhibited by Chinese older adults towards the SNH model? 4) Is there an association between the sociodemographic characteristics of Chinese older adults and their levels of expectations and acceptability concerning SNHs?

## Methods

**Fig. 1:**
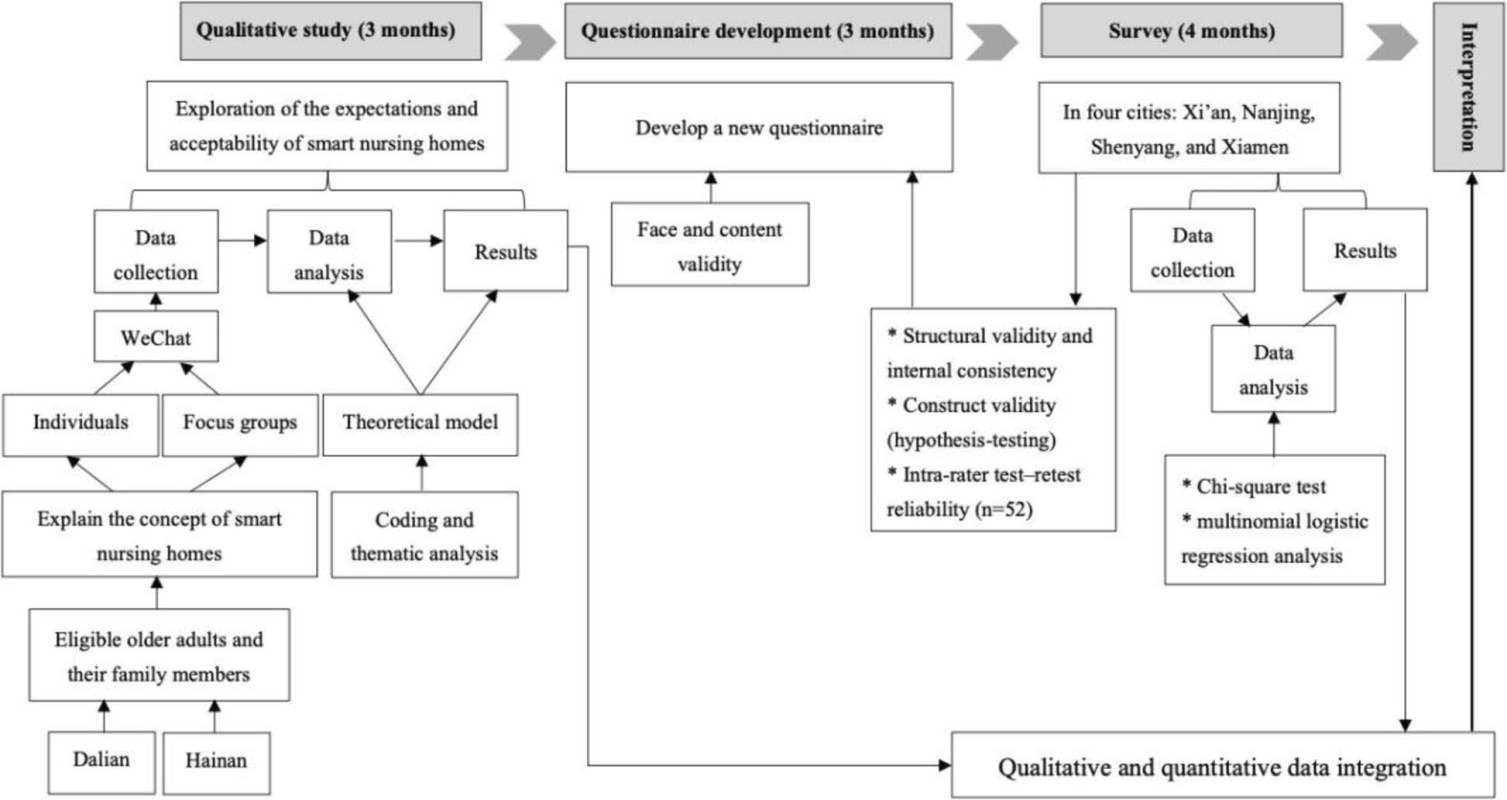
Exploratory sequential mixed methods study design

In this study, an exploratory sequential mixed method (Fig. 1) was used to answer the research questions. There were no similar instruments or pre-existing questionnaires available to measure the expectations and acceptability towards SNHs. Hence, a newly developed instrument was designed based on the results of a qualitative study to assess the levels of expectation and acceptability of SNHs among Chinese older adults. Subsequently, a survey was conducted in four Chinese cities. The sociodemographic factors associated with expectations and acceptability of SNHs were also explored and examined. Guidelines for conducting and reporting mixed research in the field of counseling and beyond guided results reporting [24] (Additional file 1). In the mixed method approach, qualitative insights were derived from a developed questionnaire assessing the expectations and acceptability. Both quantitative and qualitative data were combined in the final analysis to enhance the depth of findings. The study protocol, a scoping review and the preceding qualitative study have been previously published [10, 25, 26].

### Questionnaire development and validation

**Fig. 2:**
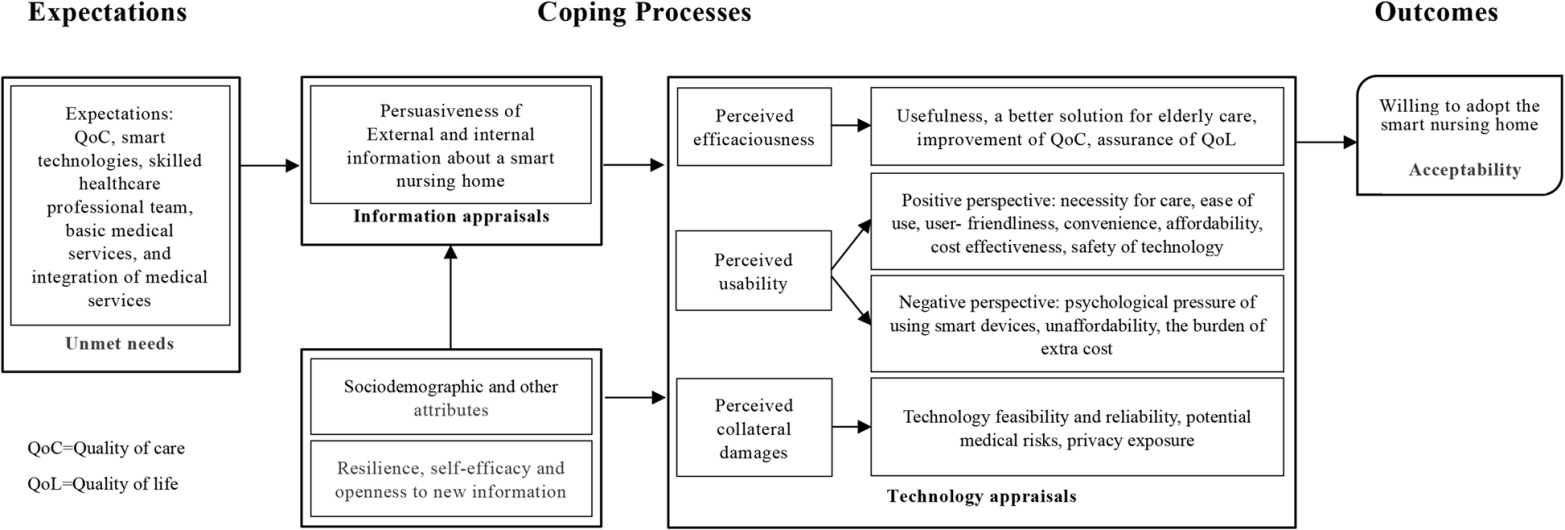
The coping process of Chinese older adults towards smart solutions in nursing homes

The questionnaire was developed as a measurement tool building on the conceptual framework (Fig. 2) derived from the ‘smart technology adoption behaviors of older consumers theory’ proposed by Golant [27], a scoping review [10], as well as the results of a qualitative study which has been published elsewhere [26]. According to the conceptual framework, the adoption of SNH emerges in response to unmet healthcare needs, resulting in unfulfilled expectations among older adults. The decision to embrace SNHs is underpinned by appraisals of information and technology. Older adults’ choices are influenced by their prior experiences with smart technologies and external sources of persuasiveness, including public media, friends, family members, and healthcare professionals (HCPs). The determinants shaping their technology appraisal encompass perceived efficaciousness, positive or negative usability, and the potential collateral damage associated with adopting smart technologies. Simultaneously, attributes specific to older adults, such as their resilience towards smart technologies, are linked to their acceptability of SNHs.

A qualitative case study was conducted using the snowball sampling method to collect data from a total of 34 participants until data saturation was achieved. Of these participants, 28 were older adults aged 60–75, residing in Hainan and Dalian, China, during the winter season. They were selected from six provinces to ensure a diverse representation of older adults. Additionally, six adult children were included in the study to explore their expectations and acceptability of SNHs. Semi-structured in-depth interviews and focus group discussions were conducted for data collection. Data were imported and managed using ATLAS.ti8 software. A framework method [28] was employed using inductive and deductive approaches to analyse the textual data. Furthermore, data were coded and categorised into themes. All items in the new questionnaire were derived from the interviews and previous scoping review through the mentioned analytical strategy. The questionnaire item design incorporated direct quotes from the qualitative data to ensure that the latter survey aligns authentically with the perspectives of the Chinese ageing population. Meanwhile, the concept of SNHs, captured from the scoping review was stated before the questionnaire to assist the respondents in sharing their perspectives on the expectation and acceptability of mart nursing homes. It included an explanation of it as a care model that provide continuous monitoring of its residents through information technologies, connect them with their remote HCPs, and integrate medical resources to satisfy the care needs of older residents. Additionally, information on sociodemographic characteristics, including age, place of residence, gender, health condition, income, type of insurance, educational attainment, number of children, and living partners, was collected from the respondents. Three items were included to measure respondents’ resilience to smart technologies, comprising familiarity with technologies, openness to new technology, and self-efficacy in applying smart technologies [27].

An expert panel, which included two statisticians, two family physicians, one public health physician, one nursing home operator, one business stakeholder, and three older adults, was invited to assess the content validity using the content validity index (CVI) for 49-item of the questionnaire [29]. This was done in line with the Consensus-based Standards for the selection of health status Measurement Instruments (COSMIN checklist) guideline [30], which evaluates the relevance, comprehensibility, and comprehensiveness of a newly developed questionnaire. Subsequently, cognitive debriefing was conducted among ten older adults [31]. Of those, eight were selected from Dalian and two from Hainan community groups. Considering the diverse characteristics of the intended respondents, three participants with primary school education were recruited, three with junior or high school education, and remaining had university education. The research team organised an online group discussion where they introduced the purpose of the study and explained the concept of SNHs, along with the content of each item in the questionnaire. Participants were instructed to provide insights into their understanding of the questions, any ambiguous terms, and potential areas of confusion. The investigator (ZYY) recorded and clarified the responses for each question. For example, the investigator used a fixed probe to ask the participants, ‘Is this a correct choice that can reflect your response? Can you paraphrase this item in your own words based on your understanding? Can you elaborate on why you chose this answer?’. The frequency of problems encountered for each question would be gathered, such as difficulties in understanding and ambiguity of wording, and adjustments would be made accordingly. One session was carried out with a duration of approximately 2–3 h.

Structural validity was established through exploratory factor analysis (EFA), based on data collected from the survey respondents. The eigenvalue was set above 1, and items with a loading value below 0.40, as well as cross-loadings greater than 0.40 were dropped [32]. Subsequently, structural equation modelling (SEM) was utilised to evaluate model fit with the SPSS AMOS software. Internal consistency was assessed using Cronbach’s alpha. A Cronbach’s alpha exceeding 0.70 is considered indicative of good internal consistency for the questionnaire [33].

Construct validity (hypothesis-testing) was assessed by comparing responses towards the expectations and acceptability of SNHs with a single item regarding willingness to move to a nursing home (Yes or No) [22]. The expectations and acceptability scores were categorised into tertiles. The hypothesis posited that the highest tertile of expectations would exhibit an association with the willingness to move to a nursing home as evidenced by an a priori odds ratio of at least 2.0, while the highest tertile of acceptability would be linked to the willingness to move to a nursing home, reflecting a priori odds ratio of at least 3.0 [34]. It was also hypothesised that expectations and acceptability would be positively correlated, with a correlation coefficient *r* value of > 0.4.

A one-month intra-rater test–retest was performed among participants who answered and returned the second completed questionnaire. The participants were recruited from those who had the willingness to participate in the test–retest and provided their telephone numbers when they answered the questionnaire for the first time.

### Quantitative study (survey)

#### Study setting

Quantitative data using surveys were collected in four major cities namely Xi’an, Nanjing, Shenyang, and Xiamen, representing the west, east, north, and south of China. In Xi’an, Nanjing, and Shenyang, the estimated older population comprises 18%, 22%, and 26%, respectively [35–37]. Meanwhile, the government of Xiamen has actively promoted smart healthcare initiatives to assist older adults in their activities of daily living [38].

#### Participants and sample size estimation

The selected older adults were within the age range of 60–75 years. Individuals residing in nursing homes, receiving palliative care, or experiencing cognitive impairment were excluded. Sample size calculation was conducted using PASS software. Based on an expected 10% level of acceptance of nursing homes among Chinese older adults [22], a 95% confidence level with a two-sided and 5% margin of error, the minimum required sample size was 139. However, for this study, a target sample size of 300 was set with inflation for non-response and incompletion rates. The data was collected from older adults who usually gather in public parks for group activities, such as morning or post-dinner exercise.

#### Data collection

A stratified random sampling method was used to identify participants. Eight enumerators (two in each city) recruited participants and asked them to suggest the ten most popular parks or communities where local older adults participate in physical activities. Subsequently, they recruited older adults from randomly selected public parks or community centres. In China, older adults typically visit public parks for collective activities, such as physical exercise and morning routines, or post-dinner dancing. Different age groups can be easily identified by the types of activities they engage in. For example, older adults aged 60–70 years usually join dancing groups, while older individuals prefer playing chess or engaging in conversations with others. Additionally, respondents were encouraged to provide their telephone numbers to enhance research credibility and facilitate participant recruitment for the intra-rater test-retest. During data collection, enumerators explained the concept of SNHs, which was stated on the questionnaire and checked the completeness of the questionnaires when all respondents returned them.

#### Data analysis

The IBM Statistical Package for Social Sciences (SPSS 26) software was used for data management and analysis. Qualitative variables were presented as frequencies and percentages. The expectations and acceptability of SNHs were categorised into tertiles.Chi-square tests were used to examine the associations among the sociodemographic factors, expectations and acceptability of SNHs, and the willingness to move to a nursing home. Multiple logistic regression models were utilised to analyse the association between the independent variables, including sociodemographic characteristics and older adults’ resilience to smart technologies, on expectations and acceptability of SNHs. Variables from the univariable regression analysis with a *p*-value < 0.20 in the expectation and acceptability domains were included in the multinomial logistic regression analysis. In all analyses, the significance level was set at 0.05. Statistical strategies to multicollinearity, data normality, and assumptions of the final model were checked.

## Results

### Questionnaire development and validation

The initial version of the questionnaire was crafted by synthesising qualitative data obtained from a scoping review and qualitative case study using both deductive and inductive analysis approaches, incorporating themes, codes, and subcodes [10, 26] (Additional file 2, A2-1). It comprised 24 items for the expectation domain, and 25 items pertaining to the acceptability domain. Among the 24 items in the expectation domain, five codes (subdomains) were identified from the qualitative phase. The subdomains are ‘quality of care supported by governments and societies’ with five items; ‘smart technology applications’ with seven items; ‘presence of a skilled HCP team’ with three items; ‘access and scope of basic medical services’ with six items; and ‘integration of medical services’ with two items. In the 25-item acceptability domain, six codes (subdomains) were identified, which encompass ‘perceived efficaciousness’ of SNHs with four items; ‘perceived positive usability’ with nine items; ‘perceived negative usability’ with two items; ‘perceived collateral damages’ with four items; ‘persuasiveness of external information’ with four items; and ‘persuasiveness of internal information’ with two items. Each item was measured on a 5-point Likert scale, where a response of 1 indicated the lowest levels of expectations or acceptability of SNHs, while a response of 5 indicated the highest levels of expectations or acceptability.

The CVI scores for relevance, comprehensibility, and comprehensiveness were 0.97, 0.96, and 0.95, respectively (Additional file 2, A2-2). These results were considered highly valuable [29]. The second version of the questionnaire had been reduced to 40 items from the initial 49 items (Additional file 2, A2-3) and named the Expectation and Acceptability of Smart Nursing Homes questionnaire (EASNH-Q). The item on willingness to move to a nursing home was moved to the sociodemographic characteristics section and all items were renumbered. All participants in the cognitive debriefing agreed with the item description and scale design for these 40 items without any problems. After undergoing the process of face and content validity, structural validity, internal consistency tests, one-month intra-rater test–retest, and construct validity were conducted using the data obtained from the latter survey among 264 respondents.

**Fig. 3:**
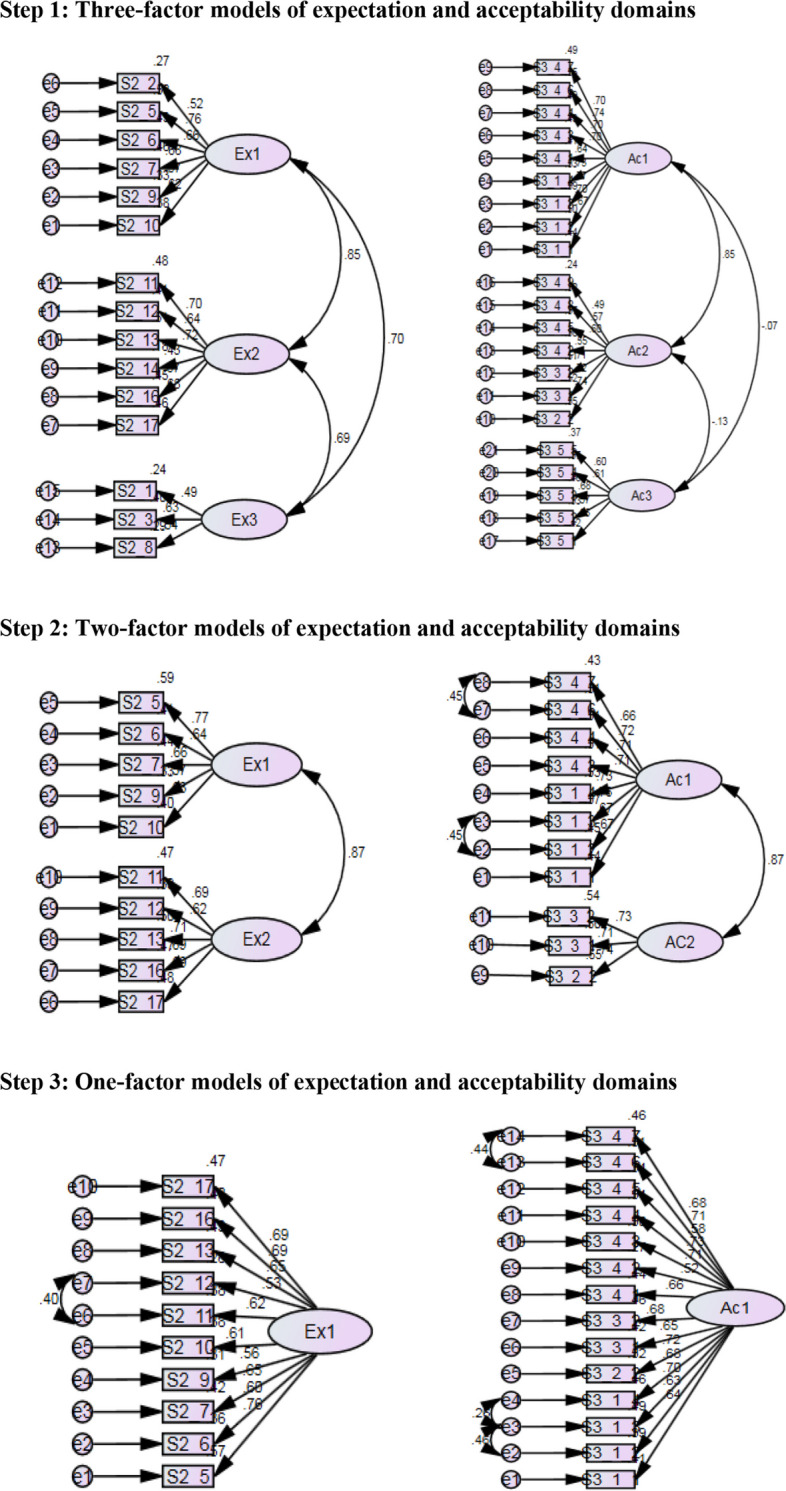
The assessment of model fit using the structural equation modelling (SEM)

EFA identified three subdomains (three factors) for the underlying structure of expectations and these three factors were renamed as nursing care, medical services, and government and social support in relation to the service categories. EFA also identified three subdomains (three factors) for the acceptability structure and the three factors were categorised as perceived usability, perceived efficaciousness, and perceived collateral damages and negative usability (Additional file 2, A2-4). In confirmatory factor analysis (CFA), single-factor models indicated the presence of 24 remaining items. Of which, 10 items in the expectation domain and 14 items in the acceptability domain were considered adequate (Table 1; Fig. 3) (Additional file 2, A2-5). Cronbach’s alpha was 0.87 in the expectation domain, and it was 0.92 in the acceptability domain.

**Table 1:** Results from confirmatory factor analyses

Domains	Model 1	Χ^2^	Df^b^	CMIN/DF^c^	CFI^d^	RMSEA^e^ (90% CI)	SRMR^f^
Expectations	Three-factor model (15 items)	186.232*^a^	87	2.14	0.92	0.07 (0.05–0.08)	0.04
	Two-factor model (10 items)	82.902*	34	2,44	0.95	0.07 (0.05–0.09)	0.04
	One-factor model (10 items)	67.947*	34	2.00	0.96	0.06 (0.04–0.08)	0.03
Acceptability	Three-factor model (21 items)	490.235	186	2.64	0.87	0.08 (0.07–0.09)	0.05
	Two-factor model (11items)	109.034*	41	2.66	0.95	0.08 (0.06-0,10)	0.04
	One-factor model (14 items)	185.450*	74	2.51	0.94	0.08 (0.06–0.09)	0.05

^a^*p < 0.05., Χ^2^ = Chi squared

^b^ *Df *Degrees of freedom

^c^ *CMIN/DF *Discrepancy divided by degree of freedom

^d^ *CFI *Comparative Fit Index

^e^ *RMSEA *Root mean square error of approximation, 90% CI = 90% confidence intervals,

^f^ *SRMR *Standardised root mean square residual

Construct validity indicated by the strong correlation between the expectations and acceptability of SNHs Pearson’s coefficient of 0.85 (*p*< 0.01). Among the 264 respondents, 84 (31.8%) were unwilling to move to nursing homes, while 180 (68.2%) expressed a willingness to move (Table 2). Type of insurance, education, the degree of familiarity with technology, openness to technology, and self-efficacy in applying smart technologies were significantly associated with the willingness to move to nursing homes. The binary logistic regression analysis for expectations and acceptability in relation to the willingness to move to nursing homes presented that the odds of older adults in the higher tertiles of expectations for SNHs towards moving to nursing homes were higher compared to those with the lowest tertile scores (OR of 1.99, 95% CI 1.01–3.93 for the middle tertile and OR of 3.02, 95% CI: 1.18–7.73 for the highest tertile) (Table 3). Similarly, the odds of older adults with the higher tertiles of acceptability for SNHs towards moving to nursing homes were higher compared to those with the lowest tertile scores. (OR of 2.36, 95% CI 1.13–4.91 for the middle terile and OR of 2.43, 95% CI: 1.11–5.39 for the highest tertile).

**Table 2:** The Association between the socioeconomic characteristics and the willingness to move to a nursing home (*n* = 264)

Variable	Total *n* (%)	No willingness to move to a NH^a^, n (%)	Having willingness to move to a NH, n (%)	*X*^*2*^ (*P* value)
Total	264 (100.0)	84 (31.8)	180 (68.2)	
**Expectations of smart NHs, 10 items (mean: 4.0, mean range: 2.0–5.0)**				
Lowest tertile (≤ 3.90)	93 (35.2)	47 (50.5)	46 (49.5)	28.89 < 0.001
Middle tertile (3.91–4.40)	101 (38.3)	29 (28.7)	72 (71.3)	
Highest tertile (≥ 4.41)	70 (26.5)	8 (11.4)	62 (88.6)	
**Acceptability of smart NHs, 14 items (mean: 4.0, mean range: 1.6–4.9)**				
Lowest tertile (≤ 3.93)	94 (35.6)	48 (51.1)	46 (48.9)	25.65 < 0.001
Middle tertile (3.94–4.29)	87 (33.0)	21 (24.1)	66 (75.9)	
Highest tertile (≥ 4.30)	83 (31.4)	15 (18.1)	68 (81.9)	
**Age**				
60–64 yo	88 (33.3)	24 (27.3)	64 (72.7)	2.20 0.333
65–70 yo	88 (33.3)	33 (37.5)	55 (62.5)	
71–75 yo	88 (33.3)	27 (30.7)	61 (69.3)	
**Gender**				
Male	130 (49.2)	40 (30.8)	90 (69.2)	0.13 0.719
Female	134 (50.8)	44 (32.8)	90 (67.2)	
**Health status**				
Healthy	98 (37.1)	36 (36.7)	62 (63.3)	1.74 0.419
One chronic disease	101 (38.3)	29 (28.7)	72 (71.3)	
Two or more chronic diseases	65 (24.6)	19 (29.2)	46 (70.8)	
**Income per month**				
No pension	30 (11.4)	11 (36.7)	19 (63.3)	2.81 0.421
1000–2000 CNY	59 (22.3)	14 (23.7)	45 (76.3)	
2000–4000 CNY	89 (33.7)	32 (36.0)	57 (64.0)	
More than 4000 CNY	86 (32.6)	27 (31.4)	59 (68.6)	
**Type of insurance**				
No insurance or with NRCMI^b^	22 (8.3)	11 (50.0)	11 (50.0)	14.85 0.002
URBMI^c^	30 (11.4)	3 (10.0)	27 (90.0)	
UEBMI^d^	181 (68.6)	65 (35.9)	116 (64.1)	
Other commercial insurance	31 (11.7)	5 (16.1)	26 (83.9)	
**Education**				
Primary school degree or lower	16 (6.1)	4 (25.0)	12 (75.0)	8.09 0.044
Junior school degree	68 (25.8)	19 (27.9)	49 (72.1)	
High school degree	139 (52.6)	54 (38.8)	85 (61.2)	
University degree or higher	41 (15.5)	7 (17.1)	34 (82.9)	
**Number of children**				
1 Child or no child	150 (56.8)	39 (26.0)	111 (74.0)	5.79 0.055
2 Children	96 (36.4)	39 (40.6)	57 (59.4)	
3 or more than 3 children	18 (6.8)	6 (33.3)	12 (66.7)	
**Living with whom**				
Alone	24 (9.1)	5 (20.8)	19 (79.2)	3.89 0.274
With partner or housemaid	125 (47.3)	44 (39.8)	81 (85.2)	
With child or children	42 (15.9)	16 (38.1)	26 (61.9)	
With partner and Children	73 (27.7)	19 (26.0)	54 (74.0)	
**Familiarity with technology**				
Not familiar with technology	54 (20.5)	34 (63.0)	20 (37.0)	31.44 < 0.001
Neutral	146 (55.3)	38 (26.0)	108 (74.0)	
Familiar with technology	64 (24.2)	12 (18.8)	52 (81.2)	
**Openness to technology**				
No (not open to technology)	76 (28.8)	50 (65.8)	26 (34.2)	56.77 < 0.001
Yes (open to technology)	188 (71.2)	34 (18.1)	154 (81.9)	
**Self-efficacy in applying smart technologies**				
No	96 (36.4)	56 (58.3)	40 (41.7)	48.89 < 0.001
Yes	168 (63.6)	28 (16.7)	140 (83.3)	

^a^ *NH *Nursing home

^b^ *NRCMI *New rural cooperative medical insurance

^c^ *URBMI *Urban resident medical insurance

^d^ *UEBMI *Urban employee basic medical insurance

**Table 3:** The association of the highest tertile expectation and acceptability of smart nursing homes and other determinants of the willingness to move to a nursing home

**Domain**	**Tertiles**	**B**	**S.E.**	**Wald**	**Sig.**	**OR**	**95% C.I.**
Expectations	Lowest			6.707			
	Middle	0.688	0.347	3.932	0.047	1.990	1.008–3.930
	Highest	1.104	0.480	5.297	0.021	3.017	1.178–7.725
**Domain**	**Variables**	**B**	**S.E.**	**Wald**	**Sig.**	**OR**	**95% C.I.**
Acceptability	Lowest			7.015			
	Middle	0.858	0.374	5.269	0.022	2.359	1.134–4.909
	Highest	0.889	0.406	4.803	0.028	2.433	1.099–5.391

In the test-retest reliability analysis, 52 participants (13 in each city) answered and returned the second completed EASNH-Q. More than half of them were women, the majority were aged 60–70. Five did not have a pension, two had no insurance, four had a primary school education, six had three or more children, and four lived alone without partners (Additional file 2, A2-6). The intraclass correlation coefficients (ICC) values for expectation and acceptability factors were 0.90 and 0.81, respectively (Additional file 2, A2-7).

### Quantitative study (survey)

In total, 264 respondents completed the questionnaires, resulting in a response rate of 70%. The demographic characteristics of the respondents are presented in Table 4. The number of respondents in each age group (60–64 years old, 65–70 years old, and 71–75 years old) was similar. Among these respondents, over 60% reported having one or more chronic diseases. More than 90% had insurance coverage and 68.1% had a high school or university education. In addition, 56.8% had one child and only 9% lived alone. Approximately one-quarter (24.2%) of the respondents were familiar with technology, 71.2% had openness to technologies, and 63.6% had self-efficacy in applying smart technologies. The overall means (SD) for expectations and acceptability were 4.0 (0.60) (Min-Max: 2.0–5.0) and 4.0 (0.60) (Min-Max: 1.6–4.9), respectively. The associations between sociodemographic characteristics and expectations and acceptability of SNHs presented that the younger age, having insurance, a university level of education, openness to technology, and self-efficacy in applying smart technologies were significantly associated with expectations (Table 5). Older age, living with partners and children, openness to technology, and self-efficacy in applying smart technologies were significantly associated with acceptability (Table 5). Table 6 displays the comparisons between the highest tertile of the expectation group and the lowest tertile of the expectation group. Older adults with self-efficacy in applying smart technologies were 28 times more likely to have the highest tertile of expectation (OR: 28.02, 95% CI: 5.92-132.66), and those with willingness to move to a nursing home were 3 times more likely to have the highest tertile of expectation (OR: 2.98, 95% CI: 1.06–8.37). Meanwhile, older adults with self-efficacy in applying smart technologies were 14 times more likely to be in the highest tertile of acceptability compared between the highest tertile of the acceptability group and the lowest tertile group (OR: 13.80, 95% CI: 4.33–43.95). The multinomial logistic regression models revealed that 41.7% (Nagelkerke R^2^ = 0.417) and 32.2% (Nagelkerke R^2^ = 0.322) of the variances in the expectation domain and the acceptability domains, respectively.

**Table 4:** Socioeconomic characteristics of the respondents(*n* = 264)

	**N(%)**
**Age**	
60–64	88 (33.3)
65–70	88 (33.3)
71–75	88 (33.3)
**Gender**	
Male	130 (49.2)
Female	134 (50.8)
**Health status**	
Healthy	98 (37.1)
One chronic disease	101 (38.3)
Two or more chronic diseases	65 (24.6)
**Income per month**	
No pension	30 (11.4)
1000–2000 CNY	59 (22.3)
2000–4000 CNY	89 (33.7)
More than 4000 CNY	86 (32.6)
**Type of insurance**	
No insurance or with NRCMI^b^	22 (8.3)
URBMI^c^	30 (11.4)
UEBMI^d^	181 (68.6)
Other commercial insurance	31 (11.7)
**Education**	
Primary school degree or lower	16 (6.1)
Junior school degree	68 (25.8)
High school degree	139 (52.6)
University degree or higher	41 (15.5)
**Number of children**	
1 Child or no child	150 (56.8)
2 Children	96 (36.4)
3 or more than 3 children	18 (6.8)
**Living with whom**	
Alone	24 (9.1)
With partner or housemaid	125 (47.3)
With child or children	42 (15.9)
With partner and Children	73 (27.7)
**Familiarity with technology**	
Not familiar with technology	54 (20.5)
Neutral	146 (55.3)
Familiar with technology	64 (24.2)
**Openness to technology**	
No (not open to technology)	76 (28.8)
Yes (open to technology)	188 (71.2)
**Self-efficacy in applying smart technologies**	
No	96 (36.4)
Yes	168 (63.6)

^a^ *NH *Nursing home

^b^ *NRCMI *New rural cooperative medical insurance

^c^ *URBMI *Urban resident medical insurance

^d^ *UEBMI *Urban employee basic medical insurance

**Table 5:** Sociodemographic characteristics of respondents according to the different categories of expectations and acceptability of smart nursing homes (*n* = 264)

**Domain**	**Variable**	**Total** **n (%)**	**Lowest tertile of expectations (Mean ≤ 3.90), n (%)**	**Middle tertile of expectations (Means: 3.91–4.40),** **n (%)**	**Highest tertile of expectations (Means ≥ 4.41), n (%)**	***X***^***2***^ **(*P*** **value)**
Expectations	Total	264 (100.0)	93 (35.2)	101 (38.3)	70 (26.5)	
	**Willingness to move to a NH** ^a^					
	No	84 (31.8)	47 (56.0)	29 (34.5)	8 (9.5)	28.885 (< 0.001)
	Yes	180 (68.2)	46 (25.6)	72 (40.0)	62 (34.4)	
	**Age**					
	60–64	88 (33.3)	21 (23.9)	37 (42.0)	30 (34.1)	14.642 (0.006)
	65–70	88 (33.3)	44 (50.0)	28 (31.8)	16 (18.2)	
	71–75	88 (33.3)	28 (31.8)	36 (40.9)	24 (27.3)	
	**Gender**					
	Male	130 (49.2)	44 (33.8)	53 (40.8)	33 (25.4)	0.684 (0.710)
	Female	134 (50.8)	49 (36.6)	48 (35.8)	37 (27.6)	
	**Health status**					
	Healthy	98 (37.1)	43 (43.9)	33 (33.7)	22 (22.4)	5.446 (0.245)
	One chronic disease	101 (38.3)	32 (31.7)	40 (39.6)	29 (28.7)	
	Two or more chronic diseases	65 (24.6)	18 (27.7)	28 (43.1)	19 (29.2)	
	**Income per month**					
	No pension	30 (11.4)	13 (43.3)	9 (30.0)	8 (26.7)	4.882 (0.559)
	1000–2000 CNY	59 (22.3)	17 (43.3)	24 (30.0)	18 (26.7)	
	2000–4000 CNY	89 (33.7)	37 (41.6)	32 (36.0)	20 (36.0)	
	More than 4000 CNY	86 (32.6)	26 (30.2)	36 (41.9)	24 (27.9)	
	**Type of insurance**					
	No insurance or with NRCMI^a^	22 (8.3)	9 (40.9)	11 (50.0)	2 (9.1)	21.412 (0.002)
	URBMI^b^	30 (11.4)	12 (40.0)	7 (23.3)	11 (36.7)	
	UEBMI^c^	181 (68.6)	65 (35.9)	76 (42.0)	40 (22.1)	
	Other commercial insurance	31 (11.7)	7 (22.6)	7 (22.6)	17 (54.8)	
	**Education**					
	Primary school degree or lower	16 (6.1)	8 (50.0)	3 (18.8)	5 (31.3)	13.388 (0.037)
	Junior school degree	68 (25.8)	22 (32.4)	24 (35.3)	22 (32.4)	
	High school degree	139 (52.6)	51 (36.7)	62 (44.6)	26 (18.7)	
	University degree or higher	41 (15.5)	12 (29.3)	12 (29.3)	17 (41.5)	
	**Number of children**					
	1 Child or no child	150 (56.8)	51 (34.0)	53 (35.3)	46 (30.7)	5.571 (0.234)
	2 Children	96 (36.4)	35 (36.5)	43 (44.8)	18 (18.8)	
	3 or more than 3 children	18 (6.8)	7 (38.9)	5 (27.8)	6 (33.3)	
	**Living with whom**					
	Alone	24 (9.1)	10 (41.7)	6 (25.0)	8 (33.3)	9.906 (0.129)
	With partner or housemaid	125 (47.3)	41 (32.8)	57 (45.6)	27 (21.6)	
	With child or children	42 (15.9)	19 (45.2)	14 (33.3)	9 (21.4)	
	With partner and Children	73 (27.7)	23 (31.5)	24 (32.9)	26 (35.6)	
	**Familiarity with technology**					
	Not familiar with technology	54 (20.5)	26 (48.1)	25 (46.3)	3 (5.6)	16.212 (0.003)
	Neutral	146 (55.3)	49 (33.6)	51 (34.9)	46 (31.5)	
	Familiar with technology	64 (24.2)	18 (28.1)	25 (39.1)	21 (32.8)	
	**Openness to technology**					
	No (not open to technology)	76 (28.8)	48 (63.2)	26 (34.2)	2 (2.6)	47.051 (< 0.001)
	Yes (open to technology)	188 (71.2)	45 (23.9)	75 (39.9)	68 (36.2)	
	**Self-efficacy in applying smart technologies**					
	No	96 (36.4)	64 (66.7)	29 (30.2)	3 (3.1)	76.011 (< 0.001)
	Yes	168 (63.6)	29 (17.3)	72 (42.9)	67 (39.9)	
**Domain**	Variable	Total n (%)	Lowest tertile of acceptability (Mean ≤ 3.93), n (%)	Middle tertile of acceptability (Means: 3.94–4.29), n (%)	Highest tertile of acceptability (Means ≥ 4.30), n (%)	***X***^***2***^ ***P*** **value**
**Acceptability**	Total	264 (100.0)	94 (35.6)	87 (33.0)	83 (31.4)	
	**Willingness to move to a nursing home**					
	No	84 (31.8)	48 (57.1)	21 (25.0)	15 (17.9)	25.644 (< 0.001)
	Yes	180 (68.2)	46 (25.6)	66 (36.7)	68 (37.8)	
	**Age**					
	60–64	88 (33.3)	23 (26.1)	32 (36.4)	33 (37.5)	9.617 (0.047)
	65–70	88 (33.3)	42 (47.7)	25 (28.4)	21 (23.9)	
	71–75	88 (33.3)	29 (33.0)	30 (34.1)	29 (33.0)	
	**Gender**					
	Male	130 (49.2)	38 (29.2)	46 (35.4)	46 (35.4)	4.651 (0.098)
	Female	134 (50.8)	56 (41.8)	41 (30.6)	37 (27.6)	
	**Health status**					
	Healthy	98 (37.1)	44 (44.9)	29 (29.6)	25 (25.5)	7.400 (0.116)
	One chronic disease	101 (38.3)	33 (32.7)	32 (31.7)	36 (35.6)	
	Two or more chronic diseases	65 (24.6)	17 (26.2)	26 (40.0)	22 (33.8)	
	**Income per month**					
	No pension	30 (11.4)	12 (40.0)	7 (23.3)	11 (36.7)	7.772 (0.255)
	1000–2000 CNY	59 (22.3)	18 (30.5)	25 (42.4)	16 (27.1)	
	2000–4000 CNY	89 (33.7)	32 (36.0)	23 (25.8)	34 (38.2)	
	More than 4000 CNY	86 (32.6)	32 (37.2)	32 (37.2)	22 (25.6)	
	**Type of insurance**					
	No insurance or with NRCMI^a^	22 (8.3)	11 (50.0)	7 (31.8)	4 (18.2)	6.523 (0.367)
	URBMI^b^	30 (11.4)	13 (43.3)	6 (20.0)	11 (36.7)	
	UEBMI^c^	181(68.6)	62 (34.3)	63 (34.8)	56 (30.9)	
	Other commercial insurance	31 (11.7)	8 (25.8)	11 (35.5)	12 (38.7)	
	**Education**					
	Primary school degree or lower	16 (6.1)	7 (43.8)	4 (25.0)	5 (31.3)	3.364 (0.762)
	Junior school degree	68 (25.8)	22 (32.4)	21 (30.9)	25 (36.8)	
	High school degree	139 (52.6)	53 (38.1)	45 (32.4)	41 (29.5)	
	University degree or higher	41 (15.5)	12 (29.3)	17 (41.5)	12 (29.3)	
	**Number of children**					
	1 Child or no child	150 (56.8)	50 (33.3)	46 (30.7)	54 (36.0)	5.208 (0.267)
	2 Children	96 (36.4)	36 (37.5)	37 (38.5)	23 (24.0)	
	3 or more than 3 children	18 (6.8)	8 (44.4)	4 (22.2)	6 (33.3)	
	**Living with whom**					
	Alone	24 (9.1)	8 (33.3)	10 (41.7)	6 (25.0)	14.149 (0.028)
	With partner or housemaid	125 (47.3)	43 (34.4)	45 (36.0)	37 (29.6)	
	With child or children	42 (15.9)	22 (52.4)	13 (31.0)	7 (16.7)	
	With partner and Children	73 (27.7)	21 (28.8)	19 (26.0)	33 (45.2)	
	**Familiarity with technology**					
	Not familiar with technology	54 (20.5)	28 (51.9)	18 (33.3)	8 (14.8)	11.442 (0.022)
	Neutral	146 (55.3)	48 (32.9)	47 (32.2)	51 (34.9)	
	Familiar with technology	64 (24.2)	18 (28.1)	22 (34.4)	24 (37.5)	
	**Openness to technology**					
	No (not open to technology)	76 (28.8)	50 (65.8)	18 (23.7)	8 (10.5)	44.936 (< 0.001)
	Yes (open to technology)	188 (71.2)	44 (23.4)	69 (36.7)	75 (39.9)	
	**Self-efficacy in applying smart technologies**					
	No	96 (36.4)	64 (66.7)	23 (24.0)	9 (9.4)	67.940 (< 0.001)
	Yes	168 (63.6)	30 (17.9)	64 (38.1)	74 (44.0)	

^a^ *NRCMI *New rural cooperative medical insurance

^b^ *URBMI *Urban resident medical insurance

^c^ *UEBMI *Urban employee basic medical insurance

**Table 6:** The multinomial logistic regression analysis of sociodemographic factors on different categories of expectations and acceptability of smart nursing homes

**Level of expectations**^**a**^	**Variables**	**Subgroups of variables**	**B**	**Std. Error**	**Wald**	**Sig.**	**OR**	**95% Confidence Interval for Exp(B) (Lower -upper bound)**	
Middle tertile of expectations (mean: 3.91–4.40)	Health status	Two or more chronic diseases	0.661	0.459	2.071	0.150	1.936	0.787	4.762
		One chronic disease	0.287	0.379	0.573	0.449	1.332	0.634	2.800
		Healthy (reference group)	0^b^	.	.	.	.	.	.
	Type of insurance	UEBMI^c^	-0.436	0.581	0.562	0.453	0.647	0.207	2.021
		URBMI^d^	-1.895	0.786	5.814	0.016	0.150	0.032	0.701
		Other commercial insurance	-0.983	0.833	1.392	0.238	0.374	0.073	1.915
		No insurance or with NRCMI^e^ (reference group)	0^b^	.	.	.	.	.	.
	Familiar with technology	Familiar with technology	-1.453	0.637	5.204	0.023	0.234	0.067	0.815
		Neutral	-1.001	0.481	4.326	0.038	0.368	0.143	0.944
		Not familiar with technology (reference group)	0^b^	.	.	.	.	.	.
	Openness to technology	Yes	0.027	0.501	0.003	0.957	1.028	0.385	2.742
		No (reference group)	0^b^	.	.	.	.	.	.
	Self-efficacy in applying smart technologies	Yes	2.272	0.504	20.353	< 0.001	9.694	3.613	26.007
		No (reference group)	0^b^	.	.	.	.	.	.
	Willingness to move to a nursing home	Yes	0.735	0.377	3.787	0.052	2.085	0.995	4.369
		No (reference group)	0^b^	.	.	.	.	.	.
Highest tertile of expectations (mean: >4.41)	Health status	Two or more chronic diseases	0.216	0.575	0.141	0.707	1.241	0.402	3.834
		One chronic disease	− 0.046	0.463	0.010	0.921	0.955	0.385	2.366
		Healthy (reference group)	0^b^	.	.	.	.	.	.
	Type of insurance	UEBMI	0.013	0.981	0.000	0.989	1.013	0.148	6.931
		URBMI	-0.545	1.090	0.251	0.617	0.580	0.068	4.905
		Other commercial insurance	0.959	1.123	0.729	0.393	2.609	0.289	23.591
		No insurance or with NRCMI (reference group)	0^b^	.	.	.	.	.	.
	Familiar with technology	Familiar with technology	-1.336	0.934	2.046	0.153	0.263	0.042	1.640
		Neutral	-0.393	0.820	0.230	0.631	0.675	0.135	3.363
		Not familiar with technology (reference group)	0^b^	.	.	.	.	.	.
	Openness to technology	Yes	1.250	0.947	1.742	0.187	3.491	0.545	22.351
		No (reference group)	0^b^	.	.	.	.	.	.
	Self-efficacy of in applying smart technologies	Yes	3.333	0.793	17.645	< 0.001	28.015	5.916	132.661
		No (reference group)	0^b^	.	.	.	.	.	.
	Willingness to move to a nursing home	Yes	1.090	0.528	4.265	0.039	2.975	1.057	8.374
		No (reference group)	0^b^	.	.	.	.	.	.
**Level of acceptability**^**f**^			**B**	**Std. Error**	**Wald**	**Sig.**	**OR**	**95% Confidence Interval for Exp(B)**	
Moderate tertile of acceptability (mean: 3.94–4.29)	Health status	Two or more chronic diseases	0.616	0.459	1.796	0.180	1.851	0.752	4.553
		One chronic disease	0.064	0.387	0.028	0.868	1.066	0.499	2.277
		Healthy (reference group)	0^b^	.	.	.	.	.	.
	Number of children	3 or more than 3 children	-0.517	0.742	0.486	0.486	0.596	0.139	2.555
		2 Children	0.278	0.372	0.558	0.455	1.321	0.637	2.741
		1 Child or no child (reference group)	0^b^	.	.	.	.	.	.
	Familiar with technology	Familiar with technology	-1.214	0.648	3.514	0.061	0.297	0.083	1.057
		Neutral	-0.783	0.493	2.525	0.112	0.457	0.174	1.201
		Not familiar with technology (reference group)	0^b^	.	.	.	.	.	.
	Openness to technology	Yes	0.508	0.513	0.981	0.322	1.661	0.608	4.538
		No (reference group)	0^b^	.	.	.	.	.	.
	Self-efficacy in applying smart technologies	Yes	1.759	0.498	12.495	< 0.001	5.808	2.190	15.406
		No (reference group)	0^b^	.	.	.	.	.	.
	Willingness to move to a nursing home	Yes	0.710	0.387	3.377	0.066	2.035	0.954	4.341
		No (reference group)	0^b^	.	.	.	.	.	.
Highest tertile of acceptability (mean: >4.30)	Health status	Two or more chronic diseases	0.797	0.510	2.444	0.118	2.218	0.817	6.022
		One chronic disease	0.308	0.411	0.564	0.453	1.361	0.609	3.043
		Healthy (reference group)	0^b^	.	.	.	.	.	.
	Number of children	3 or more than 3 children	-0.309	0.740	0.174	0.676	0.734	0.172	3.133
		2 Children	-0.329	0.417	0.621	0.431	0.720	0.318	1.631
		1 Child or no child (reference group)	0^b^	.	.	.	.	.	.
	Familiar with technology	Familiar with technology	-1.010	0.733	1.900	0.168	0.364	0.087	1.532
		Neutral	-0.405	0.604	0.449	0.503	0.667	0.204	2.179
		Not familiar with technology (reference group)	0b	.	.	.	.	.	.
	Openness to technology	Yes	0.566	0.635	0.795	0.373	1.761	0.508	6.109
		No (reference group)	0b	.	.	.	.	.	.
	Self-efficacy in applying smart technologies	Yes	2.625	0.591	19.721	< 0.001	13.801	4.333	43.954
		No (reference group)	0b	.	.	.	.	.	.
	Willingness to move to a nursing home	Yes	0.617	0.430	2.060	0.151	1.853	0.798	4.300
		No (reference group)	0^b^	.	.	.	.	.	.

^a^The reference category is the ‘lowest tertile of expectation (mean: ≤ 3.90)’

^b^ *0 *This parameter is set to zero because it is redundant

^c^ *UEBMI *Urban employee basic medical insurance

^d^ *URBMI *Urban resident medical insurance

^e^ *NRCMI *The new rural cooperative medical insurance

^f^The reference category is the ‘lowest tertile of acceptability (mean: ≤ 3.93)’

## Discussion

This is the first study in which an instrument was developed to assess the expectations and acceptability of SNHs among mainland Chinese older adults, both in general and in particular. It aims to examine their levels of expectations and acceptability towards SNHs, as well as to determine the sociodemographic factors associated with different categories of expectations and acceptability. The exploratory sequential mixed methods study design integrates various data sources offering strength to confirmatory results [39]. The study began with a qualitative phase, which explored the expectations and acceptability of a SNH model in general, and specifically among Chinese older adults and their family members. The qualitative phase mapped the knowledge bases for the development and validation of a 24-item EASNH-Q [40], and continued with a cross-sectional study in four major cities in China involving 264 respondents. Data integration was achieved through a data-building approach, in which the results from the qualitative phase and the survey were analysed and compared to understand complex phenomena, measure changes, and examine the hypothesis [24, 40]. The results from both qualitative and quantitative phases aligned with study design principles, variables exploration and analysis, and data interpretation. Many concordant findings, rather than discordant ones, were noted between the two phases. The former phase indicated the highest acceptance of moving to nursing homes as an alternative and a high level of agreeableness with external information persuasiveness for receiving healthcare benefits, such as media. A few discordant results in the later phase were related to a lower acceptance of moving to a nursing home and the family-oriented culture in healthcare decision-making as the trustworthy persuasiveness. Additionally, three items were generated from the emerging codes during the scoping review and content validity, including SNHs can provide better services to improve healthcare accessibility and availability, the preference of “human-centric” designs for the smart devices, and hospice care, were highly expected by the participants (Additional file 3).

In China, many similar questionnaires commonly focus on older adults’ willingness to move to conventional nursing homes. Two of these studies had larger samples, with 670 and 1003 Chinese older adults [22, 23], and more than half of their respondents were in aged 60–70, very similar to the main sample of this study. Additionally, more than half of the other studies’ respondents had a primary school education or lower in contrast to this study that had < 10%. In one study [22], data from an urban community showed that half of the respondents had a higher economic status which is similar to the respondents in this study (monthly pension: 1000–4000 CNY, $138–555). Regarding the proportion of willingness to move to a nursing home among Chinese older adults, this study had a higher acceptance rate (68.2%) compared to the other two previous studies (45.4–11.9%) [22, 23]. The higher acceptance rate reflects the increased demand for moving to a nursing home, particularly when older adults consider their disabilities [41]. It has been reported that older adults may choose to transition from home-based care to nursing homes with intensive supervision and more professional services due to the decline in bodily functions and the obstacles faced by family members who are unable to devote themselves to necessary or additional care [42]. As an alternative, nursing homes can provide 24-hour formal care and some medical services for older adults who require daily assistance and have complex health demands [43]. Moreover, the purpose of developing the EASNH-Q was to explore the expectations and acceptability of SNHs, making it a novel contribution. The item design of the EASNH-Q demonstrated good levels of relevance, comprehensibility, and comprehensiveness in assessing the expectations and acceptability of SNHs [44–46].

The expectations and acceptability of SNHs were explored among Chinese older adults who were interviewed in the qualitative phase. These expectations and acceptability were examined through a survey in the subsequent quantitative phase, providing empirical evidence of high levels. The survey sites selected from four different regions of mainland China represent the major group of the Chinese ageing population according to their family structures, health status, long-term care needs, and insurance schemes [47]. There were small variances in different cities when respondents answered the EASNH-Q (effect size: 0.34 − 0.32) (Additional file 4). The results showed that expectations were highly correlated with the acceptability of SNHs. Older adults from Nanjing, in the east of China, had the highest expectations of SNHs, and they also had the highest acceptability of SNHs. In contrast, older adults from Xiamen, in the south of China, had the lowest expectations and the lowest acceptability. These geographic differences among older adults may be attributed to their sociodemographic characteristics. For example, urban older adults living in environments more sustainable for an ageing population, with fewer children, higher income, and higher education have a better acceptability of nursing homes than those in rural areas who have more children, limited income, and lower education [48, 49].

In addition, the in-depth analysis of the response distribution for each item revealed that most of the questions had a ceiling effect (> 15%), except item for Q11, ‘persuasiveness of public media increases the acceptability of SNHs’ (3.8%). This reflects the report of Chinese older adults’ social network type to receive healthcare benefits, indicating that the media has less impact on appraising their health [50]. Meanwhile, the floor effect of each item was small (< 15%). The assessments of ceiling and floor effect indicate the ability of a questionnaire to distinguish among respondents at the extreme ends of the scale [51]. High ceiling effects, as observed in many of the items, may suggest a limited instrument range, measurement inaccuracy, or response bias [52]. However, no previous research has reported on the ceiling and floor effects on the expectations and acceptability of nursing homes in China. Nevertheless, the high ceiling and floor effects reflected and examined the results from the qualitative phase that all participants had a positive attitude towards SNHs [26].

It is believed that IoT, big data, and internet networks can provide quality services [53]. This belief was reflected in the responses to items Q1-5, particularly in real-time monitoring, disease prediction, electronic health records, and customised services. It is important to note that technology is not the primary reason for people deciding to move to nursing homes. Instead, technology acts as an assistant to the functions and care practices provided in nursing homes [54]. In China, more than half of older adults wish for nursing homes to provide medical services at a hospital level [22]. This study observed that many respondents had high expectations for collaboration between hospitals and SNHs to integrate medical services with remote hospitals. Moreover, Chinese older adults expected medical staff to be available at conventional nursing homes, as many nursing home residents are moderately dependent and at risk of fatal diseases [22, 55]. There were also high expectations of having trained caregivers, such as nurses and doctors in SNHs. Additionally, more than half of the respondents had high expectations of hospice care in SNHs because it is an essential part of all healthcare systems. This might be due to the general perception of the limited services and lack of accessibility of hospice care in the current nursing homes. For example, only 30.8% of nursing homes in Hebei province provided hospice care services [56].

Chinese older adults are influenced by the family-oriented culture when it comes to receiving and appraising information about their health [50]. The results were indicative of the same path that trustworthy health-related resources were typically found within family members, doctors, friends, and public media, as well as influenced by personal demands. Respondents showed a high acceptability of SNHs when they perceived the benefits and efficaciousness of using smart technologies. This perceived efficaciousness of technology generally involves a comparison between two options and the benefits received, such as comparing the quality of care and cost-effectiveness in SNHs versus conventional ones [27, 34]. Moreover, it has been commonly reported in previous studies that many older adults had negative attitudes towards adopting smart technologies due to the additional cost or the need to purchase expensive devices [14, 57, 58]. However, the high scores of items Q19-22 in the EASNH-Q confirmed that certain features of SNHs could increase older adults’ positive attitude and their consideration of adopting smart technologies. These features include the perceived necessity for health, ease of use, user-friendliness, convenience, and the “human-centric” design of smart solutions.

The final adjusted multivariable analysis showed that only self-efficacy among three items for testing the older adults’ resilience to smart technologies, including familiarity with technology and openness to technology [27], was more likely to influence the information and technology appraisals among Chinese older adults. The direct users of smart technologies designed and applied in nursing home settings have been revealed through the previous scoping review [10]. These users are nursing home residents (81%) and their HCPs (19%), such as nursing home staff and doctors in remote hospitals. Self-efficacy refers to an individual’s belief in their ability to successfully use smart technologies and older adults with self-efficacy in applying smart technologies may increase their willingness to adopt new solutions [59].

For other sociodemographic factors, such as age, income, and educational attainment, were not found to be significantly associated with the different categories of expectations and acceptability towards SNHs among Chinese older adults. These factors were previously reported in other studies to be directly associated with Chinese older adults’ willingness to move to a nursing home [22, 23], and the willingness to move to a nursing home was examined to be significantly associated with the highest tertile of expectations in this study.

This study employed several strategies to ensure research accuracy and credibility. Firstly, semi-structured, in-depth interviews, focus group discussions, and member checking were used for data collection in the qualitative study phase to ensure study credibility. A team of five investigators participated in data auditing, analysis, and coding discussions to authenticate the findings, ensuring the reliability of the study. In the quantitative phase, the survey sites chosen for data collection were selected to represent the west, east, north, and south of China. Eight onsite enumerators underwent training and were provided with a detailed study procedure to standardise the recruitment of participants and improve data quality. Data accuracy was cross-checked by the research team. However, this study has some potential limitations. Firstly, the concept of SNHs stated on the EASNH-Q was developed based on the informative literature, of which, most of the study population were from middle-income and high-income countries that may not be applicable to resource-challenged or low-income countries, as well as countries with limited internet access. Secondly, selection biases might have occurred, with qualitative study participants being Chinese older adults who were flown into Hainan and Dalian during the winter season, and quantitative study respondents coming from the four major cities [26]. This approach might not have captured all the essential factors necessary to measure the expectations and acceptability of SNHs among the entire Chinese ageing population, including other regions and rural areas in China, taking into consideration their multimorbidity and cultural differences. The findings should be generalised with caution to older adults residing in rural areas as they may have a lower acceptance of moving to a nursing home [22]. Moreover, the survey respondents in this study were selected among outdoors and able older adults, potentially missing specific groups of older people with limited mobility, economic disadvantages, or those who fall ill at home but still intend to move to nursing homes. In addition, the participants may find it difficult to answer the questions related to the acceptability of SNHs as a whole due to the non-existence of a SNH to refer to or a lack of experience using smart technologies for healthcare.

## Conclusion

The significance of this study lies in the exploration of the expectations and acceptability of SNHs among Chinese older adults, through both qualitative and quantitative evidence leading to the 24-item EASNH-Q that demonstrated commendable validity, reliability, and stability. The rigorous development process establishes it as a reliable tool for measuring the levels of expectations and acceptability of SNHs. Self-efficacy in applying smart technologies links to the high expectations and acceptability of SNHs. The willingness to relocate to a nursing home increases the high expectations of SNHs.

A feasible SNH model presents a promising solution for addressing the challenges posed by the rapidly ageing society in China. The study results hold relevance for a wide range of stakeholders and audience with an interest in SNHs, including older adults, their family members, healthcare providers, nursing home personnel, policy-makers, and entrepreneurs in the smart device industry. Furthermore, the potential applicability of these findings extends beyond China, encompassing both developed and developing nations. Subsequent research efforts should aim to quantify the expectations and acceptability of SNHs within a larger and more diverse Chinese population considering various societal strata and potentially different countries. Gaining insights from a more extensive population base will enable a more comprehensive assessment of the determinants influencing expectations and acceptability of SNHs. This, in turn, will contribute to the development of a more effective SNH model that aligns with local settings and stakeholders’ requirements.

### Supplementary Information

**Additional file 1.** The Checklist of Guidelines for Conducting and Reporting Mixed Research for Counselor Researchers.

**Additional file 2.** Questionnaire Development and Validation Process.

**Additional file 3.** The Combination and Comparison among Qualitative and Quantitative Data.

**Additional file 4.** Comparing the Variances in Cities.

## Data Availability

The dataset supporting the results and conclusions of this article is included within the article and its additional files.
