# Supplementary material for: The expectations and acceptability of a smart nursing home model among Chinese older adults: a mixed methods study
Source: BMC Nurs. 2024 Jan 13;23:40. doi: 10.1186/s12912-023-01676-0 (PMC10788001; doi:10.1186/s12912-023-01676-0)
Supplement: Supplementary file 1 — Additional file 1. The Checklist of Guidelines for Conducting and Reporting Mixed Research for Counselor Researchers. [file 12912_2023_1676_MOESM1_ESM.docx]

**Additional File 1:** The Checklist of Guidelines for Conducting and Reporting Mixed Research for Counselor Researchers

| **Sections** | **Checklist Items** | **Pages** |
| --- | --- | --- |
| **1. Research Formulation** | 1.1.1. Treat each relevant article as data that generate both qualitative (e.g., qualitative findings, literature review of source article, source article author’s conclusion) and quantitative (e.g., *p* values, effect sizes, sample size score reliability, quantitative results) information that yield a mixed research synthesis. | N/A as this is not a systematic review. |
|  | 1.1.2. Subject each document selected as part of the literature review to summarization, analysis, evaluation, and synthesis. | N/A as this is not a systematic review. |
|  | 1.1.3. Provide literature reviews that are comprehensive, current, and rigorous; that have been compared and contrasted adequately; and that contain primary sources that are relevant to the research problem under investigation, with clear connections being made between the sources presented and the present study. | Our previous scoping review. Pages 5-6, line 116-125 |
|  | 1.1.4. Present clearly the theoretical/conceptual framework. | Page 7-8 lines 158-170 |
|  | 1.1.5. Assess the findings stemming from each individual study and the emergent synthesis for trustworthiness, credibility, dependability, legitimation, validity, plausibility, applicability, consistency, neutrality, reliability, objectivity, confirmability, and/or transferability. | N/A as this is not a systematic review.  (These have been discussed in our previous scoping review and qualitative study, and used to guide and support this study) |
|  | 1.1.6. Present the goal of the study (i.e., predict; add to the knowledge base; have a personal, social, institutional, and/or organizational impact; measure change; understand complex phenomena; test new ideas; generate new ideas; inform constituencies; and examine the past). | Page 5, lines 105-108 |
|  | 1.2.1. Specify the objective(s) of the study (i.e., exploration, description, explanation, prediction, and influence). | Page 6, lines 128-131 |
|  | 1.3.1. Specify the rationale of the study. | Page 6, lines 125-128 |
|  | 1.3.2. Specify the rationale for combining qualitative and quantitative approaches (i.e., participant enrichment, instrument fidelity, treatment integrity, and significance enhancement). | Page 7, lines 144-145 |
|  | 1.4.1. Specify the purpose of the study. | See No.1.4.2. |
|  | 1.4.2. Specify the purpose for combining qualitative and quantitative approaches (e.g., identify representative sample members, conduct member check, validate individual scores on outcome measures, develop items for an instrument, identify barriers and/or facilitators within intervention condition, evaluate the fidelity of implementing the intervention and how it worked, enhance findings that are not significant, compare results from the quantitative data with the qualitative findings). | Page 7, lines 145-149 |
|  | 1.5.1. Avoid asking research questions that lend themselves to yes/no responses. | There is no such question. |
|  | 1.5.2. Present mixed research questions (i.e., questions that embed both a quantitative research question and a qualitative research question within the same question) when possible. | Page 6, lines 134-140 |
| **2. Research Planning** | 2.1.1. Specify the initial and final sample sizes for all quantitative and qualitative phases of the study. | Page 8, lines 172-177, and page 15, line 354 |
|  | 2.1.2. Present all sample size considerations made for the quantitative phase(s) (i.e., a priori power) and qualitative phases (e.g.,information-rich cases). | Page 8, lines 172-177, and page 11, lines 252-256 |
|  | 2.1.3. Present the sampling scheme for both the quantitative and qualitative phases of the study. | Page 8, lines 172-177, and page 11, line 260 |
|  | 2.1.4. Describe the mixed sampling scheme (i.e., concurrent–identical, concurrent–parallel, concurrent–nested, concurrent–multilevel, sequential–identical, sequential–parallel, sequential–nested, and sequential–multilevel). | Exploratory sequential mixed methods study design, see No. 2.1.3 |
|  | 2.1.5. Clarify the type of generalization to be made (i.e., statistical generalization, analytic generalization, and case-to-case transfer) and link it to the selected sampling design, sampling scheme, and sample size(s). | Statistical generalisation was achieved from the quantitative survey which was built on the qualitative study. Data integration was achieved through a data-building approach, in which the results from the qualitative phase and the survey were analysed and compared to understand complex phenomena, measure changes, and examine the hypothesis.  Page 17, lines 391-393 |
|  | 2.2.1. Outline the mixed research design. | Page 7, lines 145-149 |
|  | 2.2.2. Specify the quantitative research design (i.e., historical, descriptive, correlational, causal–comparative/quasi-experimental, and experimental). | Pages 10-12, lines 241-286 |
|  | 2.2.3. Specify the qualitative research design (e.g., biography, ethnographic, auto-ethnography, oral history, phenomenological, case study, grounded theory). | Page 8, lines 172-180 |
| **3. Research Implementation** | 3.1.1. Outline the mixed data collection strategy. | Page 8, lines 177-178, and page 11-12, lines 260-272 |
|  | 3.1.2. Present information about all quantitative and qualitative instruments and the process of administration. | Page 8, lines 177-178, and page 11, line 243 |
|  | 3.2.1. Outline the mixed data collection strategy (i.e., data reduction, data display, data transformation, data correlation, data consolidation, data comparison, and data integration). | Page 7, lines 151-154 |
|  | 3.2.2. Provide relevant descriptive and inferential statistics for each statistical analysis. | Page 10, lines 226-234 |
|  | 3.2.3. Discuss the extent to which the assumptions (e.g., normality, independence, equality of variances) that underlie the analyses were met, as well as any observations that might have distorted the findings (e.g., missing data, outliers). | Pages 17-18, lines 415-422, 435-438, and page 19, lines 445-456 |
|  | 3.2.4. Specify the statistical software used. | Page 12 line 275 |
|  | 3.2.5. Specify where the responsibility or authority for the creation of categories resided (i.e., participants, programs, investigative, literature, or interpretive), what the grounds were on which one could justify the existence of a given set of categories (i.e., external, rational, referential, empirical, technical, or participative), what was the source of the name used to identify a given category (i.e., participants, programs, investigative, literature, or interpretive), and at what point during the research process the categories were specified (i.e., a priori, a posteriori, or iterative). | Pages 7-8, lines 158-170, page 8, lines 178-184, page 10, lines 228-229, and page 13, lines 295-306 |
|  | 3.2.3. Specify the name of the technique used to analyze the qualitative data (e.g., content analysis method of constant comparison, discourse analysis, componential analysis, keywords in context, analytic induction, word count, domain analysis, taxonomic analysis). | Page 8, lines 178-180, page 12, lines 276-286, and our previous published qualitative study |
|  | 3.2.7. Specify the qualitative software used. | Page 8, line 178 |
|  | 3.3.1. Discuss the threats to internal validity, external validity, and measurement validity and outline the steps taken to address each of these threats to internal validity, external validity, and measurement validity. | Page 8, lines 174-175, page 9, lines 202-216, page 11, lines 243-244, and lines 260-262 |
|  | 3.3.2. Discuss the threats to trustworthiness, credibility, dependability, authenticity, verification, plausibility, applicability, confirmability, and/or transferability of data and outline all verification procedures used. | Page 21-22, lines 507-515 |
|  | 3.3.3. Discuss mixed research legitimation types (i.e., sample integration legitimation, insider–outsider legitimation, weakness minimization legitimation, sequential legitimation, conversion legitimation, paradigmatic mixing legitimation, commensurability legitimation, multiple validities legitimation, and political legitimation). | Data integration was achieved through a data-building approach, in which the results from the qualitative phase and the survey were analysed and compared to understand complex phenomena, measure changes, and examine the hypothesis. |
|  | 3.4.1. Interpret relevant types of significance of the quantitative findings (i.e., statistical significance, practical significance, clinical significance, and economic significance). | Pages 14-15, lines 330-343, and pages 15-16, lines 361-378 |
|  | 3.4.2. Conduct post hoc power analysis for all statistically nonsignificant findings. | The study achieved the intended power for the primary objective in the quantitative study. |
|  | 3.4.3. Interpret the significance (i.e., meaning) of qualitative findings. | Page 13, lines 295-304 and our previous publication for the qualitative study |
|  | 3.4.4. Discuss criteria for evaluating findings in mixed research studies (e.g., within-design consistency, conceptual consistency, interpretive agreement, interpretive distinctiveness, design suitability, design fidelity, analytic adequacy, interpretive consistency, theoretical  consistency, integrative efficacy). | Page 17, lines 391-404 |
|  | 3.5.1. Describe all steps of the mixed research process. | Pages 16-17, lines 381-393 |
|  | 3.5.2. Describe the context in which the mixed research study took place. |  |
|  | 3.5.3. Ensure that the mixed research report is accurate and complete; does not distort differences within and among individuals and groups; is free from plagiarism or misrepresentation of the ideas and conceptualizations of other scholars; and contains findings that are adequately accessible for reanalysis, further analysis, verification, or replication. | Yes |
|  | 3.5.4. Present all ethical considerations that were addressed in the study (e.g., informed consent, confidentiality, incentives, funding sources, potential conflicts of interest, biases). | Page 25-26, lines 605-612 |
|  | 3.5.5. Specify study approval in accordance with an institutional review board either in the report or in the cover letter submitted to the editor. |  |
|  | 3.5.3. Present recommendations for future research that culminate in a validation, replication, or extension of the underlying study. | Page 23, lines 548-553 |
