# Supplementary material for: The expectations and acceptability of a smart nursing home model among Chinese older adults: a mixed methods study
Source: BMC Nurs. 2024 Jan 13;23:40. doi: 10.1186/s12912-023-01676-0 (PMC10788001; doi:10.1186/s12912-023-01676-0)
Supplement: Supplementary file 2 — Additional file 2. Questionnaire Development and Validation Process. [file 12912_2023_1676_MOESM2_ESM.docx]

**Additional File 2: Questionnaire Development and Validation Process**

**A2-1: Item design for the questionnaire (49 items)**

| **Domains** | **Codes** | **Description** | **Sub-codes** | **Item development on the 1^st^ Version of EASNH-Q** |
| --- | --- | --- | --- | --- |
| **Expectations** | Extra item:  Determining the willingness to move to a nursing home | The older adults’ willingness to move to a nursing home |  | Q1. Would you like to move to a nursing home? Yes or No. |
|  | Quality of care supported by governments and societies | Chinese nursing homes are typically driven by policies rather than demand, which often results in a quality of care that fails to meet the unmet needs of older adults and their families, including access to trained caregivers, health monitoring, and assessment. Therefore, social and government support is necessary and crucial to ensure the quality of care and the future feasibility of implementing smart nursing homes. | • Government support  • Trust to the government • Social support • The cooperation of society and government | Q2. The government should take the lead in investing in the construction and operation of smart nursing homes, and other social resources should support it. |
|  |  |  | Having a clear managerial and responsible mechanism | Q3. When evaluating the service criteria of a smart nursing home, the most important criterion is that the nursing home should have a clear management mechanism and responsibility to ensure the medication and quality of life of the older adults who have dementia or require critical care. |
|  |  |  | Assurance of QoL^a^ | Q4. Safety and comfort are the most important factors in the construction of smart nursing homes.  Q5. In measuring the services of smart nursing homes, they should meet the satisfaction of the older adults or their families, or exceeds their expectations. |
|  |  |  | Effective communication | Q12. Besides using the smart technologies to provide services in the nursing homes, the effective communication is more important in daily clinical practices. This includes the effective communication among healthcare professionals, and communication between caregivers and older residents, communication between the nursing homes and the older adults’ family members. |
|  | Smart technology applications | Improving the quality of care and the accuracy of clinical practice by using effective and efficient smart solutions. | • Improving accuracy of clinical practice through advanced technologies  • Improving QoC^b^ through advanced technologies • Need more portable and smart devices to meet the medical demands of older adults | Q6. Smart nursing homes should improve the quality of care through the advanced technologies and devices. |
|  |  |  | Use of smart technologies and devices in monitoring health status and safety of older adults | Q7. Smart nursing homes should use more effective and efficient smart solutions to monitor the health status and safety of older adults and monitor them in real-time. |
|  |  |  | Risk prediction and health management | Q8. The use of smart technologies and devices in nursing homes can predict and pre-diagnose diseases for older adults, so treatment can be given at an early stage.  Q9. Smart nursing home should use smart technologies or information technologies to monitor and manage the diet and health of older adults. |
|  |  |  | Solution for emergency and first aid | Q10. Smart nursing homes should have smart facilities to monitor older adults or allow them to call caregivers when they are in a dangerous situation, so that the older residents can receive help in a timely manner. |
|  |  |  | Establishing electronic health record | Q11. The smart nursing home should have electronic health records, as it could continuously monitor the older adults health status and assist in clinical practices. |
|  |  |  | Customised care services | Q13. The older residents’ health status should be evaluated and their medical demands should be classified. Then smart nursing homes can provide the customised care services accordingly. |
|  | Presence of a skilled healthcare professional team | Healthcare professionals, in particular, general practitioners, medical specialists, trained nurses, and skilled caregivers, are expected to be available in nursing homes to provide medical services. | Medical services by healthcare professionals | Q14. The smart nursing home should have the basic medical services and the general practitioners. |
|  |  |  | Need general practitioners | Q15. The smart nursing home should have a medical room with a certain number of physicians and surgeons. |
|  |  |  | • Need specialists  • Need trained nurses and skilled caregivers | Q16. The smart nursing home should have medical specialists, trained nurses, and skilled caregivers. |
|  | Access and scope of basic medical services | A smart nursing home is expected to integrate nursing care and medical treatment to provide quality services. Such a nursing home should be built in or near a hospital to provide basic medical services. It should be able to manage chronic diseases, offer rehabilitation services, and provide programs for mental and psychological well-being. Major and emerging diseases should be treated at hospitals. This healthcare delivery model is essential for meeting the expectations of older adults and improving their overall health outcomes. | • Nursing home has the function of nursing care and medical treatment • Nursing home is built with hospital | Q17. The smart nursing home should be able to provide nursing care and medical services like a hospital.  Q23. The smart nursing home should include a hospital, or integrate with a hospital and be built with a hospital. |
|  |  |  | Nursing home is near a hospital | Q24. It is not necessary to build a smart nursing home with a hospital. It is acceptable to build a smart nursing home near a hospital so that residents can be transferred to the hospital if they need medical services. |
|  |  |  | Nursing home provides basic medical service | Q20. The smart nursing home should provide basic healthcare, but it should differ from a hospital’s services. |
|  |  |  | Major and emerging diseases are treated at hospitals and chronic diseases are managed at Nursing homes | Q18. Chronic diseases can be treated within a smart nursing home. |
|  |  |  | Rehabilitation services | Q19. The smart nursing home should have rehabilitation services. |
|  |  |  | Mental and psychological wellbeing | Q21. The smart nursing home should also consider the mental and psychological wellbeing of the older residents. |
|  | Integration of medical services | Integrating medical services from remote hospitals or remote doctors can overcome the shortage of medical resources in some rural regions and reduce unnecessary hospitalization. The integration of medical services may also facilitate seamless real-time integration between smart nursing homes and remote medical institutions. | • Integrating medical services from remote hospitals • Seamless real-time integration between smart nursing homes and remote medical institutions • Integrating with remote doctors | Q22. The smart nursing home should integrate with the remote hospitals and use their medical services or doctors in the hospitals to provide care for their residents. |
| **Acceptability** | Perceived efficaciousness | The perceived efficaciousness of smart nursing homes, including the usefulness of smart technologies, the perceived superiority of smart nursing homes as a better solution compared to traditional nursing homes or home-based care, improvements in the quality of care, and the assurance of a better quality of life, are associated with the acceptability of smart nursing homes. | Usefulness | Q27. In the technology coping process, which factors will help you make a decision to adopt smart nursing homes? Q27-1. Usefulness. |
|  |  |  | • A better solution for geriatric care • Improvement of QoC | Q27-2. Smart technologies are helpful to healthcare professionals, and it might improve their working efficiency. |
|  |  |  | Assurance of QoL | Q27-3. It is a better solution than the care provided by children or by conventional nursing homes. |
|  | Perceived usability (positive) | The perceived positive usability is associated with the acceptability of smart nursing homes, which include factors, such as the necessity for care, ease of use, user-friendliness, convenience, affordability, cost effectiveness, and safety of the technology. | Helpfulness and improvement in working efficiency^c^ | Q27-4. Smart nursing homes will ensure a better quality of life. |
|  |  |  | Improvement of healthcare accessibility and availability^c^ | Q28. In the technology coping process, which factors will help you make a decision to adopt smart nursing homes? Q28-1. Smart technologies and smart nursing homes will improve the accessibility and availability of healthcare. |
|  |  |  | Necessity for care | Q28-2. It is necessary for care. |
|  |  |  | • Ease of use  • User-friendliness | Q28-3. Ease of use and user-friendliness. |
|  |  |  | Convenience | Q28-4. Convenience. |
|  |  |  | “Human-centric” designs to fit user lifestyles^c^ | Q28-5. “Human-centric” designs that meet the demands and lifestyles of old users. |
|  |  |  | Affordability | Q28-6. Affordability. |
|  |  |  | Cost-effectiveness | Q28-7. Cost-effectiveness. |
|  |  |  | Adequate tech-support and appropriate domestication of a new technology^c^ | Q28-8. It is necessary to have adequate tech-support and appropriate domestication of a new technology. |
|  |  |  | Safety of technology | Q28-9. Ensuring the safety of technology. |
|  | Perceived usability (negative) | A negative perception towards the usability of smart solution. | Psychological pressure of using smart devices | Q29. In the technology coping process, which negative factors will affect your decision to adopt smart nursing homes? Q29-1. Psychological pressure from using smart devices, for example, feeling like your health status is serious if you are in a smart nursing home with many monitoring devices. |
|  |  |  | • Unaffordability  • Burden of extra cost | Q29-2. Unaffordability. |
|  | Perceived collateral damages | The concerns about adopting smart solutions are associated with the unintended and harmful damages of using technologies, which will result in negative user adoption. These concerns include the safety of using technologies and assaults on their lifestyles. | Feasiblity of the technology | Q30. In the technology coping process, which negative factors will affect your decision to adopt smart nursing homes? Q30-1. Feasibility and reliability of technology, for example, whether these smart technologies are feasible in operations. |
|  |  |  | Reliability of the technology | Q30-2. Concerns about potential risks in adopting smart device, for example, electrical leaks, radiation or other harm to health. |
|  |  |  | Potential medical risks | Q30-3. Lack of standards for services and supervision, for example, no official authority to standardize services provided by smart nursing homes. |
|  |  |  | Privacy exposure | Q30-4. Concerns about privacy exposure, for example, ambient monitoring devices might cause older residents to feel like their privacy has been exposure. |
|  | Persuasiveness of external information | The external information that influences the user accountability of smart solutions is received from healthcare professionals, friends, family members, and media sources. | Persuasiveness of external information from the media | Q25. In the technology coping process, which information sources will help you make a decision to adopt smart nursing homes? Q25-1. You will trust the persuasiveness of external information from the media. |
|  |  |  | Persuasiveness of external information from friends and other peers | Q25-2. You will trust the persuasiveness from friends or peers. |
|  |  |  | Persuasiveness of external information from children or family members | Q25-3. You will trust the persuasiveness from children or family members. |
|  |  |  | Persuasiveness of external information from doctors | Q25-4. You will trust the persuasiveness from doctors. |
|  | Persuasiveness of internal information | People acquire information from their past experiences and the achievement of outcomes that satisfy them. | User experience of benefit from using a new technology | Q26. In the technology coping process, which information sources will help you make a decision to adopt smart nursing homes? Q26-1. Visit smart nursing homes in person.  Q26-2. Meeting personal needs. |

^a^ QoL=Quality of life

^b^ QoC=Quality of care

^c^ Codes generated from the scoping review

**A2-2: The Score of Content Validity on Relevance Judged across the 10 Experts**

|  | **No. of the questionnaire (No. of the question)** | **Relevance (Scores from 10 examiners)** | | | | | | | | | | | | | | | **Experts in agreement** | **I-CVI^1^** | | **UA^2^** | |  |
| --- | --- | --- | --- | --- | --- | --- | --- | --- | --- | --- | --- | --- | --- | --- | --- | --- | --- | --- | --- | --- | --- | --- |
|  |  | **E1** | | | **E2** | **E3** | | **E4** | **E5** | **E6** | **E7** | **E8** | **E9** | | **E10** | |  |  |  |  |  |  |
| Section 2: Expectation of smart nursing homes | Q1 | 1 | | | 1 | 1 | | 1 | 1 | 1 | 1 | 1 | 1 | | 1 | | 10 | 1 | | 1 | |  |
|  | Q2 | 1 | | | 1 | 1 | | 1 | 1 | 1 | 1 | 1 | 1 | | 1 | | 10 | 1 | | 1 | |  |
|  | Q3 | 1 | | | 1 | 1 | | 1 | 1 | 1 | 1 | 1 | 1 | | 1 | | 10 | 1 | | 1 | |  |
|  | Q4 | 1 | | | 1 | 1 | | 1 | 1 | 1 | 1 | 1 | 1 | | 1 | | 10 | 1 | | 1 | |  |
|  | Q5 | 1 | | | 1 | 1 | | 1 | 1 | 1 | 1 | 1 | 1 | | 1 | | 10 | 1 | | 1 | |  |
|  | Q6 | 1 | | | 1 | 1 | | 1 | 1 | 1 | 1 | 1 | 1 | | 1 | | 10 | 1 | | 1 | |  |
|  | Q7 | 1 | | | 1 | 1 | | 1 | 1 | 1 | 1 | 1 | 1 | | 1 | | 10 | 1 | | 1 | |  |
|  | Q8 | 1 | | | 1 | 1 | | 1 | 1 | 1 | 1 | 1 | 0 | | 0 | | 8 | 0.8 | | 0 | |  |
|  | Q9 | 1 | | | 1 | 1 | | 1 | 1 | 1 | 1 | 1 | 0 | | 1 | | 9 | 0.9 | | 0 | |  |
|  | Q10 | 1 | | | 1 | 1 | | 1 | 1 | 1 | 1 | 1 | 1 | | 1 | | 10 | 1 | | 1 | |  |
|  | Q11 | 1 | | | 1 | 1 | | 1 | 1 | 1 | 1 | 1 | 0 | | 1 | | 9 | 0.9 | | 0 | |  |
|  | Q12 | 1 | | | 1 | 1 | | 1 | 1 | 1 | 1 | 1 | 1 | | 1 | | 10 | 1 | | 1 | |  |
|  | Q13 | 1 | | | 1 | 1 | | 1 | 1 | 1 | 1 | 1 | 0 | | 1 | | 9 | 0.9 | | 0 | |  |
|  | Q14 | 1 | | | 1 | 1 | | 1 | 1 | 1 | 1 | 1 | 1 | | 1 | | 10 | 1 | | 1 | |  |
|  | Q15 | 1 | | | 1 | 1 | | 1 | 1 | 1 | 1 | 1 | 1 | | 0 | | 9 | 0.9 | | 0 | |  |
|  | Q16 | 1 | | | 1 | 1 | | 1 | 1 | 1 | 1 | 1 | 1 | | 1 | | 10 | 1 | | 1 | |  |
|  | Q17 | 1 | | | 1 | 1 | | 1 | 1 | 1 | 1 | 1 | 1 | | 0 | | 9 | 0.9 | | 0 | |  |
|  | Q18 | 1 | | | 1 | 1 | | 1 | 1 | 1 | 1 | 1 | 1 | | 1 | | 10 | 1 | | 1 | |  |
|  | Q19 | 1 | | | 1 | 1 | | 1 | 1 | 1 | 1 | 1 | 1 | | 1 | | 10 | 1 | | 1 | |  |
|  | Q20 | 1 | | | 1 | 1 | | 1 | 1 | 1 | 1 | 1 | 1 | | 1 | | 10 | 1 | | 1 | |  |
|  | Q21 | 1 | | | 1 | 1 | | 1 | 1 | 1 | 1 | 1 | 1 | | 1 | | 10 | 1 | | 1 | |  |
|  | Q22 | 1 | | | 1 | 1 | | 1 | 1 | 1 | 1 | 1 | 1 | | 1 | | 10 | 1 | | 1 | |  |
|  | Q23 | 1 | | | 1 | 1 | | 1 | 1 | 1 | 1 | 1 | 1 | | 1 | | 10 | 1 | | 1 | |  |
|  | Q24 | 1 | | | 1 | 1 | | 1 | 1 | 1 | 1 | 1 | 1 | | 1 | | 10 | 1 | | 1 | |  |
| Section 3: Acceptability of smart nursing homes | Q25-1 | 1 | | | 1 | 1 | | 1 | 1 | 1 | 1 | 1 | 1 | | 0 | | 9 | 0.9 | | 0 | |  |
|  | Q25-2 | 1 | | | 1 | 1 | | 1 | 1 | 1 | 1 | 1 | 1 | | 1 | | 10 | 1 | | 1 | |  |
|  | Q25-3 | 1 | | | 1 | 1 | | 1 | 1 | 1 | 1 | 1 | 1 | | 1 | | 10 | 1 | | 1 | |  |
|  | Q25-4 | 1 | | | 1 | 1 | | 1 | 1 | 1 | 1 | 1 | 1 | | 1 | | 10 | 1 | | 1 | |  |
|  | Q26-1 | 1 | | | 1 | 1 | | 1 | 1 | 1 | 1 | 1 | 0 | | 1 | | 9 | 0.9 | | 0 | |  |
|  | Q26-2 | 1 | | | 1 | 1 | | 1 | 1 | 1 | 1 | 1 | 1 | | 1 | | 10 | 1 | | 1 | |  |
|  | Q27-1 | 1 | | | 1 | 1 | | 1 | 1 | 1 | 1 | 1 | 1 | | 1 | | 10 | 1 | | 1 | |  |
|  | Q27-2 | 1 | | | 1 | 1 | | 1 | 1 | 1 | 1 | 1 | 1 | | 1 | | 10 | 1 | | 1 | |  |
|  | Q27-3 | 1 | | | 1 | 1 | | 1 | 1 | 1 | 1 | 1 | 0 | | 1 | | 9 | 0.9 | | 0 | |  |
|  | Q27-4 | 1 | | | 1 | 1 | | 1 | 1 | 1 | 1 | 1 | 1 | | 1 | | 10 | 1 | | 1 | |  |
|  | Q28-1 | 1 | | | 1 | 1 | | 1 | 1 | 1 | 1 | 1 | 0 | | 1 | | 9 | 0.9 | | 0 | |  |
|  | Q28-2 | 1 | | | 1 | 1 | | 1 | 1 | 1 | 1 | 1 | 1 | | 1 | | 10 | 1 | | 1 | |  |
|  | Q28-3 | 1 | | | 1 | 1 | | 1 | 1 | 1 | 1 | 1 | 1 | | 1 | | 10 | 1 | | 1 | |  |
|  | Q28-4 | 1 | | | 1 | 1 | | 1 | 1 | 1 | 1 | 1 | 1 | | 1 | | 10 | 1 | | 1 | |  |
|  | Q28-5 | 1 | | | 1 | 1 | | 1 | 1 | 1 | 1 | 1 | 1 | | 1 | | 10 | 1 | | 1 | |  |
|  | Q28-6 | 1 | | | 1 | 1 | | 1 | 1 | 1 | 1 | 1 | 1 | | 1 | | 10 | 1 | | 1 | |  |
|  | Q28-7 | 1 | | | 1 | 1 | | 1 | 1 | 1 | 1 | 1 | 0 | | 1 | | 9 | 0.9 | | 0 | |  |
|  | Q28-8 | 1 | | | 1 | 1 | | 1 | 1 | 1 | 1 | 1 | 0 | | 1 | | 9 | 0.9 | | 0 | |  |
|  | Q28-9 | 1 | | | 1 | 1 | | 1 | 1 | 1 | 1 | 1 | 1 | | 1 | | 10 | 1 | | 1 | |  |
|  | Q29-1 | 1 | | | 1 | 1 | | 1 | 1 | 1 | 1 | 1 | 1 | | 1 | | 10 | 1 | | 1 | |  |
|  | Q29-2 | 1 | | | 1 | 1 | | 1 | 1 | 1 | 1 | 1 | 1 | | 1 | | 10 | 1 | | 1 | |  |
|  | Q30-1 | 1 | | | 1 | 1 | | 1 | 1 | 1 | 1 | 1 | 1 | | 1 | | 10 | 1 | | 1 | |  |
|  | Q30-2 | 1 | | | 1 | 1 | | 1 | 1 | 1 | 1 | 1 | 1 | | 1 | | 10 | 1 | | 1 | |  |
|  | Q30-3 | 1 | | | 1 | 1 | | 1 | 1 | 1 | 1 | 1 | 0 | | 1 | | 9 | 0.9 | | 0 | |  |
|  | Q30-4 | 1 | | | 1 | 1 | | 1 | 1 | 1 | 1 | 1 | 0 | | 1 | | 9 | 0.9 | | 0 | |  |
| Proportion relevance | | 1 | | | 1 | 1 | | 1 | 1 | 1 | 1 | 1 | 0.78 | | 0.92 | | S-CVI/Ave^3^ | 0.97 | |  | |  |
|  | |  | | |  |  | |  |  |  |  |  |  | |  | | S-CVI/UA^4^ |  | | 0.71 | |  |
| Average proportion of items judged as relevance across the ten experts | | 0.97 | | | | | | | | | | | | | | |  |  | |  | |  |
|  | **No. of the questionnaire (No. of the question)** | | **Comprehensibility (Scores from 10 examiners)** | | | | | | | | | | | | | **Experts in agreement** | | | **I-CVI** | | **UA** | |
|  |  | | **E1** | **E2** | | | **E3** | **E4** | **E5** | **E6** | **E7** | **E8** | **E9** | **E10** | |  |  |  |  |  |  |  |
| Section 2: Expectation of smart nursing homes | Q1 | | 1 | 1 | | | 1 | 1 | 1 | 1 | 1 | 1 | 1 | 1 | | 10 | | | 1 | | 1 | |
|  | Q2 | | 1 | 1 | | | 1 | 1 | 1 | 1 | 1 | 1 | 1 | 1 | | 10 | | | 1 | | 1 | |
|  | Q3 | | 1 | 1 | | | 1 | 1 | 1 | 1 | 1 | 1 | 0 | 1 | | 9 | | | 0.9 | | 0 | |
|  | Q4 | | 1 | 1 | | | 1 | 1 | 1 | 1 | 1 | 1 | 1 | 1 | | 10 | | | 1 | | 1 | |
|  | Q5 | | 1 | 1 | | | 1 | 1 | 1 | 1 | 1 | 1 | 0 | 1 | | 9 | | | 0.9 | | 0 | |
|  | Q6 | | 1 | 1 | | | 1 | 1 | 1 | 1 | 1 | 1 | 1 | 1 | | 10 | | | 1 | | 1 | |
|  | Q7 | | 1 | 1 | | | 1 | 1 | 1 | 1 | 1 | 1 | 1 | 1 | | 10 | | | 1 | | 1 | |
|  | Q8 | | 1 | 1 | | | 1 | 1 | 1 | 1 | 1 | 1 | 0 | 1 | | 9 | | | 0.9 | | 0 | |
|  | Q9 | | 1 | 1 | | | 1 | 1 | 1 | 1 | 1 | 1 | 0 | 1 | | 9 | | | 0.9 | | 0 | |
|  | Q10 | | 1 | 1 | | | 1 | 1 | 1 | 1 | 1 | 1 | 1 | 1 | | 10 | | | 1 | | 1 | |
|  | Q11 | | 1 | 1 | | | 1 | 1 | 1 | 1 | 1 | 1 | 0 | 1 | | 9 | | | 0.9 | | 0 | |
|  | Q12 | | 1 | 1 | | | 1 | 1 | 1 | 1 | 1 | 1 | 0 | 1 | | 9 | | | 0.9 | | 0 | |
|  | Q13 | | 1 | 1 | | | 1 | 1 | 1 | 1 | 1 | 1 | 0 | 1 | | 9 | | | 0.9 | | 0 | |
|  | Q14 | | 1 | 1 | | | 1 | 1 | 1 | 1 | 1 | 1 | 0 | 1 | | 9 | | | 0.9 | | 0 | |
|  | Q15 | | 1 | 1 | | | 1 | 1 | 1 | 1 | 1 | 1 | 1 | 1 | | 10 | | | 1 | | 1 | |
|  | Q16 | | 1 | 1 | | | 1 | 1 | 1 | 1 | 1 | 1 | 1 | 1 | | 10 | | | 1 | | 1 | |
|  | Q17 | | 1 | 1 | | | 1 | 1 | 1 | 1 | 1 | 1 | 0 | 1 | | 9 | | | 0.9 | | 0 | |
|  | Q18 | | 1 | 1 | | | 1 | 1 | 1 | 1 | 1 | 1 | 0 | 1 | | 9 | | | 0.9 | | 0 | |
|  | Q19 | | 1 | 1 | | | 1 | 1 | 1 | 1 | 1 | 1 | 1 | 1 | | 10 | | | 1 | | 1 | |
|  | Q20 | | 1 | 1 | | | 1 | 1 | 1 | 1 | 1 | 1 | 0 | 1 | | 9 | | | 0.9 | | 0 | |
|  | Q21 | | 1 | 1 | | | 1 | 1 | 1 | 1 | 1 | 1 | 1 | 1 | | 10 | | | 1 | | 1 | |
|  | Q22 | | 1 | 1 | | | 1 | 1 | 1 | 1 | 1 | 1 | 1 | 1 | | 10 | | | 1 | | 1 | |
|  | Q23 | | 1 | 1 | | | 1 | 1 | 1 | 1 | 1 | 1 | 1 | 1 | | 10 | | | 1 | | 1 | |
|  | Q24 | | 1 | 1 | | | 1 | 1 | 1 | 1 | 1 | 1 | 1 | 1 | | 10 | | | 1 | | 1 | |
| 问卷第三部分：智能养老院接受度 (Section 3: Acceptability of smart nursing homes) | Q25-1 | | 1 | 1 | | | 1 | 1 | 1 | 1 | 1 | 1 | 1 | 1 | | 10 | | | 1 | | 1 | |
|  | Q25-2 | | 1 | 1 | | | 1 | 1 | 1 | 1 | 1 | 1 | 1 | 1 | | 10 | | | 1 | | 1 | |
|  | Q25-3 | | 1 | 1 | | | 1 | 1 | 1 | 1 | 1 | 1 | 1 | 1 | | 10 | | | 1 | | 1 | |
|  | Q25-4 | | 1 | 1 | | | 1 | 1 | 1 | 1 | 1 | 1 | 1 | 1 | | 10 | | | 1 | | 1 | |
|  | Q26-1 | | 1 | 1 | | | 1 | 1 | 1 | 1 | 1 | 1 | 1 | 1 | | 10 | | | 1 | | 1 | |
|  | Q26-2 | | 1 | 1 | | | 1 | 1 | 1 | 1 | 1 | 1 | 1 | 1 | | 10 | | | 1 | | 1 | |
|  | Q27-1 | | 1 | 1 | | | 1 | 1 | 1 | 1 | 1 | 1 | 1 | 1 | | 10 | | | 1 | | 1 | |
|  | Q27-2 | | 1 | 1 | | | 1 | 1 | 1 | 1 | 1 | 1 | 0 | 1 | | 9 | | | 0.9 | | 0 | |
|  | Q27-3 | | 1 | 1 | | | 1 | 1 | 1 | 1 | 1 | 1 | 0 | 1 | | 9 | | | 0.9 | | 0 | |
|  | Q27-4 | | 1 | 1 | | | 1 | 1 | 1 | 1 | 1 | 1 | 1 | 1 | | 10 | | | 1 | | 1 | |
|  | Q28-1 | | 1 | 1 | | | 1 | 1 | 1 | 1 | 1 | 1 | 0 | 1 | | 9 | | | 0.9 | | 0 | |
|  | Q28-2 | | 1 | 1 | | | 1 | 1 | 1 | 1 | 1 | 1 | 1 | 1 | | 10 | | | 1 | | 1 | |
|  | Q28-3 | | 1 | 1 | | | 1 | 1 | 1 | 1 | 1 | 1 | 1 | 1 | | 10 | | | 1 | | 1 | |
|  | Q28-4 | | 1 | 1 | | | 1 | 1 | 1 | 1 | 1 | 1 | 1 | 1 | | 10 | | | 1 | | 1 | |
|  | Q28-5 | | 1 | 1 | | | 1 | 1 | 1 | 1 | 1 | 1 | 0 | 1 | | 9 | | | 0.9 | | 0 | |
|  | Q28-6 | | 1 | 1 | | | 1 | 1 | 1 | 1 | 1 | 1 | 1 | 1 | | 10 | | | 1 | | 1 | |
|  | Q28-7 | | 1 | 1 | | | 1 | 1 | 1 | 1 | 1 | 1 | 0 | 1 | | 9 | | | 0.9 | | 0 | |
|  | Q28-8 | | 1 | 1 | | | 1 | 1 | 1 | 1 | 1 | 1 | 0 | 1 | | 9 | | | 0.9 | | 0 | |
|  | Q28-9 | | 1 | 1 | | | 1 | 1 | 1 | 1 | 1 | 1 | 0 | 1 | | 9 | | | 0.9 | | 0 | |
|  | Q29-1 | | 0 | 1 | | | 1 | 1 | 1 | 1 | 1 | 1 | 0 | 1 | | 8 | | | 0.8 | | 0 | |
|  | Q29-2 | | 1 | 1 | | | 1 | 1 | 1 | 1 | 1 | 1 | 1 | 1 | | 10 | | | 1 | | 1 | |
|  | Q30-1 | | 1 | 1 | | | 1 | 1 | 1 | 1 | 1 | 1 | 1 | 1 | | 10 | | | 1 | | 1 | |
|  | Q30-2 | | 1 | 1 | | | 1 | 1 | 1 | 1 | 1 | 1 | 1 | 1 | | 10 | | | 1 | | 1 | |
|  | Q30-3 | | 1 | 1 | | | 1 | 1 | 1 | 1 | 1 | 1 | 0 | 1 | | 9 | | | 0.9 | | 0 | |
|  | Q30-4 | | 1 | 1 | | | 1 | 1 | 1 | 1 | 1 | 1 | 1 | 1 | | 10 | | | 1 | | 1 | |
| Proportion relevance | | | 0.98 | 1 | | | 1 | 1 | 1 | 1 | 1 | 1 | 0.6 | 1 | | S-CVI/Ave | | | 0.96 | | 0.59 | |
|  | | |  |  | | |  |  |  |  |  |  |  |  | | S-CVI/UA | | |  | |  | |
| Average proportion of items judged as relevance across the ten experts | | | 0.96 | | | | | | | | | | | | |  | | |  | |  | |

|  | **No. of the questionnaire (No. of the question)** | **Comprehensiveness (Scores from 10 examiners)** | | | | | | | | | | **Experts in agreement** | **I-CVI** | **UA** |
| --- | --- | --- | --- | --- | --- | --- | --- | --- | --- | --- | --- | --- | --- | --- |
|  |  | **E1** | **E2** | **E3** | **E4** | **E5** | **E6** | **E7** | **E8** | **E9** | **E10** |  |  |  |
| Section 2: Expectation of smart nursing homes | Q1 | 1 | 1 | 1 | 1 | 1 | 1 | 1 | 1 | 0 | 1 | 9 | 0.9 | 0 |
|  | Q2 |  |  |  |  |  |  |  |  |  |  |  |  |  |
|  | Q3 |  |  |  |  |  |  |  |  |  |  |  |  |  |
|  | Q4 |  |  |  |  |  |  |  |  |  |  |  |  |  |
|  | Q5 |  |  |  |  |  |  |  |  |  |  |  |  |  |
|  | Q6 |  |  |  |  |  |  |  |  |  |  |  |  |  |
|  | Q7 |  |  |  |  |  |  |  |  |  |  |  |  |  |
|  | Q8 |  |  |  |  |  |  |  |  |  |  |  |  |  |
|  | Q9 |  |  |  |  |  |  |  |  |  |  |  |  |  |
|  | Q10 |  |  |  |  |  |  |  |  |  |  |  |  |  |
|  | Q11 |  |  |  |  |  |  |  |  |  |  |  |  |  |
|  | Q12 |  |  |  |  |  |  |  |  |  |  |  |  |  |
|  | Q13 |  |  |  |  |  |  |  |  |  |  |  |  |  |
|  | Q14 |  |  |  |  |  |  |  |  |  |  |  |  |  |
|  | Q15 |  |  |  |  |  |  |  |  |  |  |  |  |  |
|  | Q16 |  |  |  |  |  |  |  |  |  |  |  |  |  |
|  | Q17 |  |  |  |  |  |  |  |  |  |  |  |  |  |
|  | Q18 |  |  |  |  |  |  |  |  |  |  |  |  |  |
|  | Q19 |  |  |  |  |  |  |  |  |  |  |  |  |  |
|  | Q20 |  |  |  |  |  |  |  |  |  |  |  |  |  |
|  | Q21 |  |  |  |  |  |  |  |  |  |  |  |  |  |
|  | Q22 |  |  |  |  |  |  |  |  |  |  |  |  |  |
|  | Q23 |  |  |  |  |  |  |  |  |  |  |  |  |  |
|  | Q24 |  |  |  |  |  |  |  |  |  |  |  |  |  |
| Section 3: Acceptability of smart nursing homes | Q25-1 | 1 | 1 | 1 | 1 | 1 | 1 | 1 | 1 | 1 | 1 | 10 | 1 | 1 |
|  | Q25-2 |  |  |  |  |  |  |  |  |  |  |  |  |  |
|  | Q25-3 |  |  |  |  |  |  |  |  |  |  |  |  |  |
|  | Q25-4 |  |  |  |  |  |  |  |  |  |  |  |  |  |
|  | Q26-1 |  |  |  |  |  |  |  |  |  |  |  |  |  |
|  | Q26-2 |  |  |  |  |  |  |  |  |  |  |  |  |  |
|  | Q27-1 |  |  |  |  |  |  |  |  |  |  |  |  |  |
|  | Q27-2 |  |  |  |  |  |  |  |  |  |  |  |  |  |
|  | Q27-3 |  |  |  |  |  |  |  |  |  |  |  |  |  |
|  | Q27-4 |  |  |  |  |  |  |  |  |  |  |  |  |  |
|  | Q28-1 |  |  |  |  |  |  |  |  |  |  |  |  |  |
|  | Q28-2 |  |  |  |  |  |  |  |  |  |  |  |  |  |
|  | Q28-3 |  |  |  |  |  |  |  |  |  |  |  |  |  |
|  | Q28-4 |  |  |  |  |  |  |  |  |  |  |  |  |  |
|  | Q28-5 |  |  |  |  |  |  |  |  |  |  |  |  |  |
|  | Q28-6 |  |  |  |  |  |  |  |  |  |  |  |  |  |
|  | Q28-7 |  |  |  |  |  |  |  |  |  |  |  |  |  |
|  | Q28-8 |  |  |  |  |  |  |  |  |  |  |  |  |  |
|  | Q28-9 |  |  |  |  |  |  |  |  |  |  |  |  |  |
|  | Q29-1 |  |  |  |  |  |  |  |  |  |  |  |  |  |
|  | Q29-2 |  |  |  |  |  |  |  |  |  |  |  |  |  |
|  | Q30-1 |  |  |  |  |  |  |  |  |  |  |  |  |  |
|  | Q30-2 |  |  |  |  |  |  |  |  |  |  |  |  |  |
|  | Q30-3 |  |  |  |  |  |  |  |  |  |  |  |  |  |
|  | Q30-4 |  |  |  |  |  |  |  |  |  |  |  |  |  |
| Proportion relevance | | 1 | 1 | 1 | 1 | 1 | 1 | 1 | 1 | 0.5 | 1 | S-CVI/Ave | 0.95 |  |
|  | |  |  |  |  |  |  |  |  |  |  | S-CVI/UA |  | 0.5 |
| Average proportion of items judged as relevance across the ten experts | | 0.95 | | | | | | | | | |  |  |  |

^1^ I-CVI=Item-level content validity index

^2^ UA=Universal agreement

^3^ S-CVI/Ave=Scare-level content validity index based on the average method

^4^ S-CVI/UA=Scare-level content validity index based on the universal agreement method

**A2-3: The Second Version of EASNH-Q (40 Items)** **after Face and Content Validity**

| **1^st^ Version** | **2^nd^ Version after Face and Content Validity** |
| --- | --- |
| **Expectations (24 Items)** | **Expectations (18 Items)** |
| Q1. Would you like to move to a nursing home? | Moved to the section on sociodemographic characteristics |
| Q2. The government should take the lead in investing in the construction and operation of smart nursing homes, and other social resources should support it. | S2_1. Smart nursing homes should be led and implemented by the government. |
| Q3. When evaluating the service criteria of a smart nursing home, the most important criterion is that the nursing home should have a clear management mechanism and responsibility to ensure the medication and quality of life of the older adults who have dementia or require critical care. | S2_2. Smart nursing homes should have a clear management mechanism to guarantee the quality of care for the older residents. |
| Q4. Safety and comfort are the most important factors in the construction of smart nursing homes. | Deleted |
| Q5. In measuring the services of smart nursing homes, they should meet the satisfaction of the older adults or their families, or exceeds their expectations. | Deleted |
| Q6. Smart nursing homes should improve the quality of care through the advanced technologies and devices. | S2_4. Smart nursing homes should improve the quality of care by using more smart technologies. |
| Q7. Smart nursing homes should use more effective and efficient smart solutions to monitor the health status and safety of older adults and monitor them in real-time. | S2_5. The smart nursing home should use more effective/efficient smart solution to monitor the elderly people in real time. |
| Q8. The use of smart technologies and devices in nursing homes can predict and pre-diagnose diseases for older adults, so treatment can be given at an early stage. | S2_6. Smart nursing homes should use smart technologies to predict the risk of disease for the elderly residents |
| Q9. Smart nursing home should use smart technologies or information technologies to monitor and manage the diet and health of older adults. | S2_7. The smart nursing home should use smart technologies to monitor and manage diet for the elderly residents. |
| Q10. Smart nursing homes should have smart facilities to monitor older adults or allow them to call caregivers when they are in a dangerous situation, so that the older residents can receive help in a timely manner. | S2_8. Smart nursing homes should have some facilities for emergency issues so that the older residents can reach the medical staff in time. |
| Q11. The smart nursing home should have electronic health records, as it could continuously monitor the older adults’ health status and assist in clinical practices. | S2_9. The smart nursing home should have electronic health records to manage the health for their elderly residents in a long-term. |
| Q12. Besides using the smart technologies to provide services in the nursing homes, the effective communication is more important in daily clinical practices. This includes the effective communication among healthcare professionals, and communication between caregivers and older residents, communication between the nursing homes and the older adults’ family members. | S2_3. Effective communication between people is crucial to ensure the quality of care, such as effective communication among healthcare professionals, communication between caregivers and older residents, and communication between nursing homes and the older adults’ family members. |
| Q13. The older residents’ health status should be evaluated and their medical demands should be classified. Then smart nursing homes can provide the customised care services accordingly. | S2_10. Smart nursing homes should use smart technologies to assess and analyse the health of the elderly people in order to provide the customised services. |
| Q14. The smart nursing home should have the basic medical services and the general practitioners. | S2_11. The qualified doctors should be available in the smart nursing home. |
| Q15. The smart nursing home should have a medical room with a certain number of physicians and surgeons. | Deleted |
| Q16. The smart nursing home should have medical specialists, trained nurses and professional caregivers. | S2_12. Smart nursing homes should be staffed by professionally trained caregivers. |
| Q17. The smart nursing home should be able to provide nursing care and medical services like a hospital. | S2_13. The smart nursing home should attach a hospital to provide the medical services. |
| Q18. Chronic diseases can be treated within a smart nursing home. | Deleted |
| Q19. The smart nursing home should have rehabilitation services. | S2_15. Smart nursing homes should have rehabilitation services. |
| Q20. The smart nursing home should provide basic healthcare, but it should differ from a hospital’s services. | S2_14. Smart nursing homes should be built near a hospital s that older residents can receive medical treatment easily. |
| Q21. The smart nursing home should also consider the mental and psychological wellbeing of the older residents. | S2_18. Smart nursing homes should also consider the mental and psychological wellbeing of the older residents. |
| Q22. The smart nursing home should integrate with the remote hospitals and use their medical services or doctors in the hospitals to provide care for their residents. | S2_16. The smart nursing home should integrate with medical facilities or doctors from the remote hospitals to provide care for their residents. |
| Q23. The smart nursing home should include a hospital, or integrate with a hospital and be built with a hospital. | Merged to S2-13 |
| Q24. It is not necessary to build a smart nursing home with a hospital. It is acceptable to build a smart nursing home near a hospital so that residents can be transferred to the hospital if they need medical services. | Merged to S2-14 |
|  | **New item was suggested by nursing home stakeholder S2_17. The hospice care should be provided in smart nursing homes.** |
| **Acceptability (25 Items)** | **Acceptability (22 Items)** |
| Q25. In the technology coping process, which information sources will help you make a decision to adopt smart nursing homes? Q25-1. You will trust the persuasiveness of external information from the media. | S3_1. What information sources will influence your acceptance of smart nursing homes? S3_1_1. The persuasiveness of public media. |
| Q25-2. You will trust the persuasiveness from friends or peers. | S3_1_2. The persuasiveness of friends or the peers. |
| Q25-3. You will trust the persuasiveness from children or family members. (会听信儿女或家人所说的。) | S3_1_3. The persuasiveness of children or family members. |
| Q25-4. You will trust the persuasiveness from doctors. | S3_1_4. The persuasiveness of doctors. |
| Q26. In the technology coping process, which information sources will help you make a decision to adopt smart nursing homes? Q26-1. Visit smart nursing homes in person. | S3_2. What experiences will help you make a decision to adopt the smart nursing homes? S3_2_1. Visiting smart nursing homes in person. |
| Q26-2. Meeting personal needs. | S3_2_2. Meeting personal needs. |
| Q27. In the technology coping process, which factors will help you make a decision to adopt smart nursing homes? Q27-1. Usefulness. | S3_3. What factors will motivate your acceptability of smart nursing homes? S3_3_1. Smart nursing homes offer better services than traditional nursing. |
| Q27-2. Smart technologies are helpful to healthcare professionals, and it might improve their working efficiency. | S3_3_2. Smart technologies can improve the efficiency of healthcare professionals. |
| Q27-3. It is a better solution than the care provided by children or by conventional nursing homes. | Merged to S3_3_1. |
| Q27-4. Smart nursing homes will ensure a better quality of life. | Merged to S3_3_1. |
| Q28. In the technology coping process, which factors will help you make a decision to adopt smart nursing homes? Q28-1. Smart technologies and smart nursing homes will improve the accessibility and availability of healthcare. | S3_4. Which factors will help you make a decision to adopt smart nursing homes? S3_4_1. Smart technologies and smart nursing homes can provide better services for the elderly residents. |
| Q28-2. It is necessary for care. | S3_4_2. It is necessary for care. |
| Q28-3. Ease of use and user-friendliness. | S3_4_3. Ease of use and user-friendly. |
| Q28-4. Convenience. | S3_4_4. Convenience to carry on or use. |
| Q28-5. “Human-centric” designs that meet the demands and lifestyles of old users. | S3_4_5. “Human-centric” designs to fit the demands of the elderly user. |
| Q28-6. Affordability. | S3_4_6. Affordability. |
| Q28-7. Cost-effectiveness. | S3_4_7. Cost effectiveness for its services. |
| Q28-8. It is necessary to have adequate tech-support and appropriate domestication of a new technology. | S3_4_8. It is necessary to have adequate tech-support and appropriate domestication of a new technology. |
| Q28-9. Ensuring the safety of technology. | S3_4_9. Ensuring the safety of technology. |
| Q29. In the technology coping process, which negative factors will affect your decision to adopt smart nursing homes? Q29-1. Psychological pressure from using smart devices, for example, feeling like your health status is serious if you are in a smart nursing home with many monitoring devices. | S3_5. Which negative factors will affect your decision to adopt the smart nursing homes? S3_5_1. Psychological pressure from using smart devices, for example, feeling like your health status is serious if you are in a smart nursing home with many monitoring devices. |
| Q29-2. Unaffordability. | Deleted |
| Q30. In the technology coping process, which negative factors will affect your decision to adopt smart nursing homes? Q30-1. Feasibility and reliability of technology, for example, whether these smart technologies are feasible in operations. | S3_5_2. Feasibility of the smart nursing homes, for example, whether these smart technologies are feasible in operations. |
| Q30-2. Concerns about potential risks in adopting smart device, for example, electrical leaks, radiation or other harm to health. | S3_5_3. Concerns about potential risks in adopting smart device, for example, electrical leaks, radiation or other harms to the health. |
| Q30-3. Lack of standards for services and supervision, for example, no official authority to standardize services provided by smart nursing homes. | S3_5_4. Lack of standards for services provided by smart nursing homes and supervision. |
| Q30-4. Concerns about privacy exposure, for example, ambient monitoring devices might cause older residents to feel like their privacy has been exposure. | S3_5_5. Concerns about privacy exposure. |

**A2-4: Item Descriptive Statistics and Rotated Factor Loadings from EFA**

| **Items** | **Mean** | **SD** | **Factor loadings** | | |
| --- | --- | --- | --- | --- | --- |
| **Expectation domain** |  |  | **Nursing care** | **Medical services** | **Government and social support** |
| S2_2. Smart nursing homes should have a clear management mechanism to guarantee the quality of care for the older residents. | 4.1 | 0.76 | 0.613 | -0.017 | 0.337 |
| The smart nursing home should use more effective/efficient smart solution to monitor the elderly people in real time | 3.8 | 1.07 | 0.734 | 0.351 | 0.093 |
| S2_6. Smart nursing homes should use smart technologies to predict the risk of disease for the elderly residents. | 4.1 | 0.81 | 0.678 | 0.123 | 0.301 |
| S2_7. The smart nursing home should use smart technologies to monitor and manage diet for the elderly residents. | 3.9 | 0.95 | 0.551 | 0.349 | 0.286 |
| S2_9. The smart nursing home should have electronic health records to manage the health for their elderly residents in a long-term. | 4.1 | 0.76 | 0.606 | 0.269 | 0.064 |
| S2_10. Smart nursing homes should use smart technologies to assess and analyse the health of the elderly people in order to provide the customised services. | 4.2 | 0.81 | 0.595 | 0.329 | 0.082 |
| S2_11. The qualified doctors should be available in the smart nursing home. | 4.3 | 0.73 | 0.184 | 0.628 | 0.444 |
| S2_12. Smart nursing homes should be staffed by professionally trained caregivers. | 4.2 | 0.71 | 0.058 | 0.658 | 0.448 |
| S2_13. The smart nursing home should attach a hospital to provide the medical services. | 4.1 | 0.77 | 0.216 | 0.711 | 0.268 |
| S2_14. Smart nursing homes should be built near a hospital s that older residents can receive medical treatment easily. | 4.0 | 0.77 | 0.21 | 0.519 | -0.011 |
| S2_16. The smart nursing home should integrate with medical facilities or doctors from the remote hospitals to provide care for their residents. | 3.9 | 0.87 | 0.482 | 0.513 | 0.133 |
| S2_17. The hospice care should be provided in smart nursing homes. | 3.6 | 1.17 | 0.447 | 0.686 | -0.114 |
| S2_1. Smart nursing homes should be led and implemented by the government. | 4.0 | 0.70 | 0.175 | 0.018 | 0.685 |
| S2_3. Effective communication between people is crucial to ensure the quality of care, such as effective communication among healthcare professionals, communication between caregivers and older residents, and communication between nursing homes and the older adults’ family members. | 4.2 | 0.76 | 0.280 | 0.144 | 0.605 |
| S2_8. Smart nursing homes should have some facilities for emergency issues so that the older residents can reach the medical staff in time. | 4.3 | 0.72 | 0.071 | 0.189 | 0.690 |
| KMO = 0.903, Bartlett's test χ2 = 1307.114, p < 0.001 | | | | | |
| **Items** | **Mean** | **SD** | **Factor loadings** | | |
| **Acceptability domain** | **Mean** | **SD** | **Perceived usability** | **Perceived efficaciousness** | **Perceived collateral damages and negative usability** |
| S3_1_1. The persuasiveness of public media. | 3.3 | 0.92 | 0.665 | 0.232 | -0.104 |
| S3_1_2. The persuasiveness of friends or the peers. | 3.7 | 0.88 | 0.835 | 0.014 | 0.039 |
| S3_1_3. The persuasiveness of children or family members. | 4.2 | 0.88 | 0.845 | 0.107 | 0.024 |
| S3_1_4. The persuasiveness of doctors. | 4.0 | 0.93 | 0.753 | 0.196 | -0.100 |
| S3_4_1. Smart technologies and smart nursing homes can provide better services for the elderly residents. | 4.0 | 0.67 | 0.543 | 0.416 | -0.037 |
| S3_4_3. Ease of use and user-friendly. | 4.1 | 0.86 | 0.568 | 0.449 | -0.015 |
| S3_4_4. Convenience to carry on or use. | 4.0 | 0.89 | 0.587 | 0.440 | -0.027 |
| S3_4_6. Affordability. | 4.1 | 0.91 | 0.702 | 0.305 | -0.004 |
| S3_4_7. Cost effectiveness for its services. | 4.2 | 0.91 | 0.571 | 0.458 | 0.129 |
| S3_2_2. Meeting personal needs. | 4.2 | 0.90 | 0.497 | 0.589 | 0.156 |
| S3_3_1. Smart nursing homes offering better services than conventional nursing homes. | 4.0 | 0.77 | 0.410 | 0.593 | -0.114 |
| S3_3_2. Smart technologies improving the efficiency of healthcare professionals. | 4.1 | 0.81 | 0.462 | 0.540 | -0.131 |
| S3_4_2. It is necessary for care. | 4.1 | 0.83 | 0.263 | 0.573 | -0.057 |
| S3_4_5. “Human-centric” designs to fit the demands of the elderly user. | 4.1 | 0.81 | 0.384 | 0.507 | -0.020 |
| S3_4_8. It is necessary to have adequate tech-support and appropriate domestication of a new technology. | 4.1 | 0.79 | 0.156 | 0.684 | -0.131 |
| S3_4_9. Ensuring the safety of technology. | 4.4 | 0.71 | 0.020 | 0.730 | -0.015 |
| S3_5_1. Psychological pressure from using smart devices, for example, feeling like your health status is serious if you are in a smart nursing home with many monitoring devices. | 3.6 | 0.84 | -0.152 | 0.134 | 0.746 |
| S3_5_2. Feasibility of the smart nursing homes, for example, whether these smart technologies are feasible in operations. | 3.9 | 0.81 | -0.009 | -0.235 | 0.644 |
| S3_5_3. Concerns about potential risks in adopting smart device, for example, electrical leaks, radiation or other harms to the health. | 3.8 | 0.93 | 0.108 | -0.084 | 0.751 |
| S3_5_4. Lack of standards for services provided by smart nursing homes and supervision. | 3.9 | 0.78 | 0.147 | -0.243 | 0.677 |
| S3_5_5. Concerns about privacy exposure. | 3.6 | 1.00 | -0.200 | 0.209 | 0.721 |
| KMO = 0.907, Bartlett's test χ2 = 2429.808, p < 0.001 | | | | | |

**A2-5: The Final Version of EASNH-Q (24 Items)**

**The expectation and acceptability of smart nursing homes questionnaire (EASNH-Q)**

(Hello! This questionnaire is an academic questionnaire based on the PhD project of Zhao Yuanyuan at the Universiti Putra Malaysia. It takes approximately 5-10 minutes to complete this questionnaire. Your information and name will be remained confidential. If you have any questions or comments, please contact Zhao Yuanyuan (helenzhao78@qq.com). We would like to thank you in advance for your participation in the research.)

**(Consent Form)**

1. (The Chinese elderly people’s expectations and acceptability to a smart nursing home model: A mixed methods study)

2. (Please answer the questionnaire. All questions only have one answer.)

3. (Site and samples: This study will be limited to Xi'an, Shenyang, Nanjing and Xiamen. The target population is the elderly people aged 60-75 years old. The exclusion criteria also includes: 1) the elderly people who have already moved in a nursing home; 2) with predicting life expectancy of less than one year in clinical practice; 3) with diagnostic psychiatric disorders that may impair ability in answering or completing questionnaires.)

4. (The benefit of participation: The participant will be given a gift after completes the questionnaires. Gifts will be delivered to participants’ homes directly. For more details, the investigator will explain to each participant.)

6. (Participating in the research is not anticipated to cause you any disadvantages or discomfort. The potential physical and/or psychological harm or distress will be the same as any experienced in everyday life. Participants are free to decide for participation, and the decision may not jeopardise the opportunity to receive any services/assistance from the government.)

7. (Any personal information during the academic publication or other academic outlets that could identify yourself is not revealed; These anonymised data will not allow any individuals or their institutions to be identified or identifiable.)

(This project has been ethically approved by the Ethics Committee for Research Involving Human Subjects Universiti Putra Malaysia and Hainan Medical University, China. If you have any complaints about the project in the first instance you can contact one of our investigators. If you feel your complaint has not been handled to your satisfaction you can contact the Universiti Putra Malaysia and Hainan Medical University, China to take your complaint further.)

(The project is a doctoral thesis that conducted by Zhao, Yuanyuan at Universiti Putra Malaysia. The researcher(s) received no financial support for the research, and future publication of this research.)

(If you have read and understood the above, please sign and continue to answer the questionnaire. Thank you.)

(Name)： (Tel)：

(Date)：

**(Page 1)**

**(Section 1: Demographic characteristics and other information)**

| 1.  Your age range | 1）60-64 years old；2）65-70 years old；3）71-75 years old. |
| --- | --- |
| 2.  Residential place | 1）Xi’an；2）Nanjing；3）Shenyang；4）Xiamen |
| 3.  Gender | 1）Male；2）female |
| 4.  Health condition | 1）More than 2 chronic diseases（such as cardiovascular diseases, diabetes, Hyperlipidemia, cancer, asthma etc.）；2）one chronic disease；3）healthy. |
| 5.  Income range | 1）No income/no pension；2）1000-2000 CNY；3）2000-4000 CNY；4）more than 4000 CNY |
| 6.  Types of insurance | 1）No insurance；2）The new rural cooperative medical insurance (NRCMI)； 3）Urban resident medical insurance (URBMI)； 4）Urban Employee Basic Medical Insurance (UEBMI)；5）Other commercial insurance. |
| 7.  Level of education | 1）Primary school degree or lower；2）junior school degree；3）high school degree；4）university degree or higher |
| 8.  Number of children | 1）No Child；2）1 child；3）2 children；4）3 or more than 3 children. |
| 9.  Living with others | 1）Alone；2）With partner；3）With Child or Children；4）With partner and Children；5）With others (i.e: housemaid） |
| 10.  Are you familiar with using smart phone, computer or other electronic products? | 1）Not at all；2）not familiar with；3）neutral；4）familiar with. |
| 11.  Are you interest in adopting smart technologies? | 1）I am not interest；2）I am interest. |
| 12.  Are you willing to solve the difficulty during applying smart technologies? | 1）No, I won’t；2）Yes, I will. |
| 13.  Do you have willingness to move to a nursing home? | 1）No；2）Yes |

(The concept of smart nursing homes for your reference)

(A smart nursing home as a collective or individual senior care model that integrates tending of life routines and healthcare needs of its residents with information technologies or engineering that may provide continuous monitoring to its residents, connected communication within its care providers, and teleconsultation with external medical resources. Technology-assisted nursing care ensures life enjoyment in an affordable and safe environment, and immediate health attention with people-centered care that is effective, efficient, and evidence-based.)

Sample explanation

(Smart nursing homes use technologies such as information engineering and the Internet of Things to provide high quality, efficient, safe and personalised monitoring services for the elderly people, ensuring that they can enjoy a relatively free life in a safe environment. Specifically, smart nursing homes install sensors in rooms, mattresses or clothing to automatically collect and analyse the health indicators, such as heartbeat, body temperature, nighttime sleep and other physical states and biological data such as gait, which can predict diseases in advance, prevent falls, track the mentally challenged elderly people and prevent wandering. The integration of medical services in nursing homes makes use of remote hospitals and specialists to maximise the provision of medical services to the elderly people in nursing homes. At the same time, nursing homes digitally manage the health records of the elderly people, making it easier for doctors or nurses to manage treatment and rehabilitation. Family members can also remotely monitored their parents’ daily life in the nursing home, and can check their health status via mobile phones or the Internet. In addition, nursing homes use a number of assistive facilities such as robots or smart devices to assist with nursing care.)

**(Section 2: Expectations of smart nursing homes)**

| （Questions） | Strongly disagree | Disagree | Neutral | Agree | Strongly Agree |
| --- | --- | --- | --- | --- | --- |
| Q1 (S2_5).  (The smart nursing home should use more effective/efficient smart solution to monitor the elderly people in real time.) | 1 | 2 | 3 | 4 | 5 |
| Q2 (S2_6).  (Smart nursing homes should use smart technologies to predict the risk of disease for the elderly residents.) | 1 | 2 | 3 | 4 | 5 |
| Q3 (S2_7).  (The smart nursing home should use smart technologies to monitor and manage diet for the elderly residents.) | 1 | 2 | 3 | 4 | 5 |
| Q4 (S2_9).  (The smart nursing home should have electronic health records to manage the health for their elderly residents in a long-term.) | 1 | 2 | 3 | 4 | 5 |
| Q5 (S2_10).  (Smart nursing homes should use smart technologies to assess and analyse the health of the elderly people in order to provide the customised services.) | 1 | 2 | 3 | 4 | 5 |
| （Questions） | Low expected | Unexpected | Neutral | Expected | Very expected |
| Q6 (S2_11).  (The qualified doctors should be available in the smart nursing home.) | 1 | 2 | 3 | 4 | 5 |
| Q7 (S2_12).  (Smart nursing homes should be staffed by professionally trained caregivers.) | 1 | 2 | 3 | 4 | 5 |
| Q8 (S2_13).  (The smart nursing home should attach a hospital to provide the medical services.) | 1 | 2 | 3 | 4 | 5 |
| Q9 (S2_16).  (The smart nursing home should integrate with medical facilities or doctors from the remote hospitals to provide care for their residents.) | 1 | 2 | 3 | 4 | 5 |
| Q10 (S2_17).  (The hospice care should be provided in smart nursing homes.) | 1 | 2 | 3 | 4 | 5 |

**(Section 3: Acceptability of smart nursing homes)**

| （Questions） | Strongly untrustful | Untrustful | Neutral | Trustful | Strongly trustful |
| --- | --- | --- | --- | --- | --- |
| Q11 (S3_1_1).  (The persuasiveness of public media.) | 1 | 2 | 3 | 4 | 5 |
| Q12 (S3_1_2).  (The persuasiveness of friends or the peers.) | 1 | 2 | 3 | 4 | 5 |
| Q13 (S3_1_3).  (The persuasiveness of children or family members.) | 1 | 2 | 3 | 4 | 5 |
| Q14 (S3_1_4).  (The persuasiveness of doctors.) | 1 | 2 | 3 | 4 | 5 |
| （Questions） | Strongly disagree | Disagree | Neutral | Agree | Strongly Agree |
| Q15 (S3_2_2).  (Meeting personal needs.) | 1 | 2 | 3 | 4 | 5 |
| Q16 (S3_3_1).  (Smart nursing homes offer better services than traditional nursing.) | 1 | 2 | 3 | 4 | 5 |
| Q17 (S3_3_2).  (Smart technologies can improve the efficiency of healthcare professionals.) | 1 | 2 | 3 | 4 | 5 |
| Q18 (S3_4_1).  (Smart technologies and smart nursing homes can provide better services for the elderly residents.) | 1 | 2 | 3 | 4 | 5 |
| Q19 (S3_4_2).  (It is necessary for care.) | 1 | 2 | 3 | 4 | 5 |
| Q20 (S3_4_3).  (Ease of use and user-friendly.) | 1 | 2 | 3 | 4 | 5 |
| Q21 (S3_4_4).  (Convenience to carry on or use.) | 1 | 2 | 3 | 4 | 5 |
| Q22 (S3_4_5).  (“Human-centric” designs to fit the demands of the elderly user.) | 1 | 2 | 3 | 4 | 5 |
| Q23 (S3_4_6).  (Affordability.) | 1 | 2 | 3 | 4 | 5 |
| Q24 (S3_4_7).  (Cost effectiveness for its services.) | 1 | 2 | 3 | 4 | 5 |

**(Signature of on-site investigator)：**

**(Site)：**

**(Duration of answering the questionnaire)： （Minute）**

**A2-6: The Socioeconomic Characteristics of the participants for One-month Intra-rater Test–retest (n=52)**

| **Age** | **N(%)** | ^a^ NH=Nursing home  ^b^ NRCMI= New rural cooperative medical.  insurance  ^c^ URBMI= Urban resident medical insurance  ^d^ UEBMI= Urban employee basic medical  insurance |
| --- | --- | --- |
| 60-64 | 19 (36.5) |  |
| 65-70 | 21 (40.4) |  |
| 71-75 | 12 (23.1) |  |
| **Gender** |  |  |
| Male | 21 (40.4) |  |
| Female | 31 (59.6) |  |
| **Health status** |  |  |
| Healthy | 18 (34.6) |  |
| One chronic disease | 21 (40.4) |  |
| Two or more chronic diseases | 13 (25.0) |  |
| **Income per month** |  |  |
| No pension | 5 (9.6) |  |
| 1000-2000 CNY | 15 (28.8) |  |
| 2000-4000 CNY | 16 (30.8) |  |
| More than 4000 CNY | 16 (30.8) |  |
| **Type of insurance** |  |  |
| No insurance or with NRCMI^b^ | 2 (3.8) |  |
| URBMI^c^ | 7 (13.5) |  |
| UEBMI^d^ | 34 (65.4) |  |
| Other commercial insurance | 9 (17.3) |  |
| **Education** |  |  |
| Primary school degree or lower | 4 (7.7) |  |
| Junior school degree | 11 (21.2) |  |
| High school degree | 29 (55.8) |  |
| University degree or higher | 8 (15.4) |  |
| **Number of children** |  |  |
| 1 Child or no child | 31 (59.6) |  |
| 2 Children | 15 (28.8) |  |
| 3 or more than 3 children | 6 (11.5) |  |
| **Living with whom** |  |  |
| Alone | 4 (7.7) |  |
| With partner or housemaid | 27 (51.9) |  |
| With child or children | 8 (15.4) |  |
| With partner and Children | 13 (25.0) |  |
| **Familiarity with technology** |  |  |
| Not familiar with technology | 6 (11.5) |  |
| Neutral | 33 (63.5) |  |
| Familiar with technology | 13 (25.0) |  |
| **Openness to technology** |  |  |
| No  (not open to technology) | 13 (25.0) |  |
| Yes  (open to technology) | 39 (75.0) |  |
| **Self-efficacy** **in applying smart technologies** |  |  |
| No | 16 (30.8) |  |
| Yes | 36 (69.2) |  |

**A2-7: Total Score of One-month Intra-rater Test–retest Reliability (n=52)**

| **N=52** | **Test (T1)** | **Retest (T2)** | **ICC**  ***(p-*value*)*** | **95% CI** |
| --- | --- | --- | --- | --- |
|  | **Mean (SD)** | **Mean (SD)** |  |  |
| Expectations of smart nursing homes | 4.0 (0.67) | 4.1 (0.56) | 0.896  (*p*<0.001) | 0.819-0.940 |
| Acceptability of smart nursing homes | 4.0 (0.70) | 4.2 (0.42) | 0.809  (*p*<0.001) | 0.667-0.890 |
