# Supplementary material for: The expectations and acceptability of a smart nursing home model among Chinese older adults: a mixed methods study
Source: BMC Nurs. 2024 Jan 13;23:40. doi: 10.1186/s12912-023-01676-0 (PMC10788001; doi:10.1186/s12912-023-01676-0)
Supplement: Supplementary file 3 — Additional file 3. The Combination and Comparison among Qualitative and Quantitative Data. [file 12912_2023_1676_MOESM3_ESM.docx]

**Additional File 3:** **The Combination and Comparison among Qualitative and Quantitative Data**

| **The Results of the Qualitative Study** | **The Results of the Quantitative Study (n=264)** | | | | | |
| --- | --- | --- | --- | --- | --- | --- |
| **Responses from the interviewed participants and the number of the participants commented**  **(n=34)** | **Items/Scores** | **1** | **2** | **3** | **4** | **5** |
| Use of smart technologies and devices in monitoring health status and safety of the elderly people (60.7%, 17 EP^a^; 66.7%, 4 FM^b^) | Q1 (S2_5).  (The smart nursing home should use more effective/efficient smart solution to monitor the elderly people in real time.) | 3.8% (10/264) | 9.5% (25/264) | 17.4% (46/264) | 41.7% (110/264) | 27.7% (73/264) |
| Risk prediction and health management (25.0%, 7 EP; 33.3%, 2 FM) | Q2 (S2_6).  (Smart nursing homes should use smart technologies to predict the risk of disease for the elderly residents.) | 0% (0/264) | 3.4% (9/264) | 18.9% (50/264) | 43.6% (115/264) | 34.1% (90/264) |
|  | Q3 (S2_7).  (The smart nursing home should use smart technologies to monitor and manage diet for the elderly residents.) | 1.1% (3/264) | 8.7% (23/264) | 17.8% (47/264) | 43.6% (115/264) | 28.8% (76/264) |
| Solution for emergency and first aid (25.0%, 7 EP; 50.0%, 3 FM) | Q4 (S2_9).  (The smart nursing home should have electronic health records to manage the health for their elderly residents in a long-term.) | 0% (0/264) | 1.5% (4/264) | 11.7% (31/264) | 44.7% (118/264) | 42.0% (111/264) |
| Establishing electronic health record (3.6%, 1 EP; 16.7%, 1 FM) | Q5 (S2_10).  (Smart nursing homes should use smart technologies to assess and analyse the health of the elderly people in order to provide the customised services.) | 0.4% (1/264) | 3.8% (10/264) | 10.6% (28/264) | 43.6% (115/264) | 41.7% (110/264) |
| Medical services and doctors (32.1%, 9 EP)  Need general practitioners (21.4%, 6 EP)  Need physicians and surgeons（7.1%, 2 EP） | Q6 (S2_11).  (The qualified doctors should be available in the smart nursing home.) | 0% (0/264) | 1.5% (4/264) | 11.7% (31/264) | 44.7% (118/264) | 42.0% (111/264) |
| Need medical specialists, trained nurses and, skilled caregivers (32.1%, 9 EP; 16.7%, 1 FM) | Q7 (S2_12).  (Smart nursing homes should be staffed by professionally trained caregivers.) | 0% (0/264) | 2.7% (7/264) | 8.3% (22/264) | 53.0% (140/264) | 36.0% (95/264) |
| Nursing home has the function of nursing care and medical treatment (21.4%, 6 EP)  Nursing home is built with hospital (7.1%, 2 EP; 16.7%, 1 FM) | Q8 (S2_13).  (The smart nursing home should attach a hospital to provide the medical services.) | 0% (0/264) | 4.9% (13/264) | 12.1% (32/264) | 54.9% (145/264) | 28.0% (74/264) |
| Integrating medical services from remote hospitals (50.0%, 14 EP; 66.7%, 4 FM)  Seamless real time integration between smart nursing homes and remote medical institutions (10.7%, 3 EP)  Integrating with remote doctors (10.7%, 3 EP; 16.7%, 1 FM) | Q9 (S2_16).  (The smart nursing home should integrate with medical facilities or doctors from the remote hospitals to provide care for their residents.) | 0% (0/264) | 6.4% (17/264) | 22.7% (60/264) | 43.2% (114/264) | 27.7% (73/264) |
| Emerging code proposed by expert panel | Q10 (S2_17).  (The hospice care should be provided in smart nursing homes.) | 8.0% (21/264) | 9.5% (25/264) | 17.0% (45/264) | 42.8% (113/264) | 22.7% (60/264) |
| Persuasiveness of external information from the media (3.6%, 1 EP; 33.3%, 2 FM) | Q11 (S3_1_1).  (The persuasiveness of public media.) | 4.9% (13/264) | 13.3% (35/264) | 31.4% (83/264) | 46.6% (123/264) | 3.8% (10/264) |
| Persuasiveness of external information from friends and other peers (10.7%, 3 EP; 16.7%, 1 FM) | Q12 (S3_1_2).  (The persuasiveness of friends or the peers.) | 1.9% (5/264) | 8.0% (21/264) | 20.1% (53/264) | 54.9% (145/264) | 15.2% (40/264) |
| Persuasiveness of external information from children or family members (14.3%, 4 EP; 33.3%, 2 FM) | Q13 (S3_1_3).  (The persuasiveness of children or family members.) | 0.8% (2/264) | 4.2% (11/264) | 14.0% (37/264) | 39.0% (103/264) | 42.0% (111/264) |
| Persuasiveness of external information from doctors (10.7%, 3 EP; 16.7%, 1 FM) | Q14 (S3_1_4).  (The persuasiveness of doctors.) | 1.5% (4/264) | 5.3% (14/264) | 17.8% (47/264) | 42.4% (112/264) | 33.0% (87/264) |
| User experience of benefit from using a new technology (3.6%, 1 EP; 50.0%, 3 FM) | Q15 (S3_2_2).  (Meeting personal needs.) | 1.9% (5/264) | 3.8% (10/264) | 8.0% (21/264) | 40.9% (108/264) | 45.5% (120/264) |
| Usefulness (32.1%, 9 EP; 50.0%, 3 FM) | Q16 (S3_3_1).  (Smart nursing homes offer better services than traditional nursing.) | 0% (0/264) | 5.3% (14/264) | 12.9% (34/264) | 56.4% (149/264) | 25.4% (67/264) |
| A better solution for geriatric care (3.6%, 1 EP)  Improvement of quality of care (3.6%, 1 EP; 16.7%, 1 FM) | Q17 (S3_3_2).  (Smart technologies can improve the efficiency of healthcare professionals.) | 0.4% (1/264) | 4.2% (11/264) | 15.2% (40/264) | 50.8% (134/264) | 29.5% (78/264) |
| Improvement of healthcare accessibility and availability (Additional code from our scoping review) | Q18 (S3_4_1).  (Smart technologies and smart nursing homes can provide better services for the elderly residents.) | 0.4% (1/264) | 2.3% (6/264) | 16.3% (43/264) | 64.4% (170/264) | 16.7% (44/264) |
| Necessity for care (67.9%, 19 EP; 50.0%, 3 FM) | Q19 (S3_4_2).  (It is necessary for care.) | 0.8% (2/264) | 3.0% (8/264) | 16.3% (43/264) | 45.8% (121/264) | 34.1% (90/264) |
| Ease of use (17.9%, 5 EP; 33.3%, 2 FM)  User-friendly (3.6%, 1 EP; 16.7%, 1 FM) | Q20 (S3_4_3).  (Ease of use and user-friendly.) | 0% (0/264) | 5.7% (15/264) | 17.8% (47/264) | 42.4% (112/264) | 34.1% (90/264) |
| Convenience (21.4%, 6 EP; 50.0%, 3 FM) | Q21 (S3_4_4).  (Convenience to carry on or use.) | 1.1% (3/264) | 5.7% (15/264) | 15.9% (42/264) | 47.3% (125/264) | 29.9% (79/264) |
| Human-centric” designs to fit user lifestyles (Additional code from our scoping review) | Q22 (S3_4_5).  ( “Human-centric” designs to fit the demands of the elderly user.) | 0.4% (1/264) | 4.5% (12/264) | 12.5% (33/264) | 51.1% (135/264) | 31.4% (83/264) |
| Affordability (75.0%, 21 EP; 83.3%, 5 FM) | Q23 (S3_4_6).  (Affordability.) | 1.9% (5/264) | 5.3% (14/264) | 11.4% (30/264) | 47.3% (125/264) | 34.1% (90/264) |
| Cost effectiveness (emerging code) (10.7%, 3 EP; 33.3%, 2 FM) | Q24 (S3_4_7).  (Cost effectiveness for its services.) | 1.1% (3/264) | 5.3% (14/264) | 11.0% (29/264) | 39.0% (103/264) | 43.6% (115/264) |

^a^ EP= Mentioned frequency from elderly people (total 28 elderly people)

^b^ FM= Mentioned frequency from family members (total 6 family members)
