# Supplementary material for: The expectations and acceptability of a smart nursing home model among Chinese older adults: a mixed methods study
Source: BMC Nurs. 2024 Jan 13;23:40. doi: 10.1186/s12912-023-01676-0 (PMC10788001; doi:10.1186/s12912-023-01676-0)
Supplement: Supplementary file 4 — Additional file 4. Comparing the Variances in Cities. [file 12912_2023_1676_MOESM4_ESM.docx]

**Additional File 4: Comparing the Variances in Cities**

**A: Comparing the variances of expectations of smart nursing homes in cities**

|  |  | **N** | **Mean** | **Std. Deviation** | **Std. Error** | **95% Confidence Interval for Mean** |  | **Minimum** | **Maximum** |
| --- | --- | --- | --- | --- | --- | --- | --- | --- | --- |
|  |  |  |  |  |  | Lower Bound | Upper Bound |  |  |
| Number of expectation items | Xi'an | 66 | 1.80 | 0.533 | 0.066 | 1.67 | 1.93 | 1 | 3 |
|  | Nanjing | 66 | 2.52 | 0.588 | 0.072 | 2.37 | 2.66 | 1 | 3 |
|  | Shenyang | 66 | 2.08 | 0.865 | 0.106 | 1.86 | 2.29 | 1 | 3 |
|  | Xiamen | 66 | 1.26 | 0.506 | 0.062 | 1.13 | 1.38 | 1 | 3 |
|  | Total | 264 | 1.91 | 0.782 | 0.048 | 1.82 | 2.01 | 1 | 3 |
| Q1^1^ (S2_5)^2^ | Xi'an | 66 | 4.09 | 0.799 | 0.098 | 3.89 | 4.29 | 1 | 5 |
|  | Nanjing | 66 | 4.44 | 0.558 | 0.069 | 4.30 | 4.58 | 3 | 5 |
|  | Shenyang | 66 | 3.92 | 0.917 | 0.113 | 3.70 | 4.15 | 1 | 5 |
|  | Xiamen | 66 | 2.74 | 1.071 | 0.132 | 2.48 | 3.01 | 1 | 5 |
|  | Total | 264 | 3.80 | 1.065 | 0.066 | 3.67 | 3.93 | 1 | 5 |
| Q2 (S2_6) | Xi'an | 66 | 4.05 | 0.666 | 0.082 | 3.88 | 4.21 | 3 | 5 |
|  | Nanjing | 66 | 4.55 | 0.532 | 0.065 | 4.41 | 4.68 | 3 | 5 |
|  | Shenyang | 66 | 4.02 | 0.903 | 0.111 | 3.79 | 4.24 | 2 | 5 |
|  | Xiamen | 66 | 3.73 | 0.887 | 0.109 | 3.51 | 3.95 | 2 | 5 |
|  | Total | 264 | 4.08 | 0.814 | 0.050 | 3.98 | 4.18 | 2 | 5 |
| Q3 (S2_7) | Xi'an | 66 | 3.94 | 0.762 | 0.094 | 3.75 | 4.13 | 2 | 5 |
|  | Nanjing | 66 | 4.38 | 0.576 | 0.071 | 4.24 | 4.52 | 3 | 5 |
|  | Shenyang | 66 | 3.98 | 0.984 | 0.121 | 3.74 | 4.23 | 1 | 5 |
|  | Xiamen | 66 | 3.30 | 1.095 | 0.135 | 3.03 | 3.57 | 1 | 5 |
|  | Total | 264 | 3.90 | 0.954 | 0.059 | 3.79 | 4.02 | 1 | 5 |
| Q4 (S2_9) | Xi'an | 66 | 4.15 | 0.614 | 0.076 | 4.00 | 4.30 | 3 | 5 |
|  | Nanjing | 66 | 4.44 | 0.530 | 0.065 | 4.31 | 4.57 | 3 | 5 |
|  | Shenyang | 66 | 4.29 | 0.799 | 0.098 | 4.09 | 4.48 | 2 | 5 |
|  | Xiamen | 66 | 3.70 | 0.841 | 0.103 | 3.49 | 3.90 | 1 | 5 |
|  | Total | 264 | 4.14 | 0.757 | 0.047 | 4.05 | 4.24 | 1 | 5 |
| Q5 (S2_10) | Xi'an | 66 | 4.15 | 0.588 | 0.072 | 4.01 | 4.30 | 3 | 5 |
|  | Nanjing | 66 | 4.79 | 0.412 | 0.051 | 4.69 | 4.89 | 4 | 5 |
|  | Shenyang | 66 | 4.21 | 0.832 | 0.102 | 4.01 | 4.42 | 2 | 5 |
|  | Xiamen | 66 | 3.74 | 0.950 | 0.117 | 3.51 | 3.98 | 1 | 5 |
|  | Total | 264 | 4.22 | 0.813 | 0.050 | 4.12 | 4.32 | 1 | 5 |
| Q6 (S2_11) | Xi'an | 66 | 4.23 | 0.576 | 0.071 | 4.09 | 4.37 | 3 | 5 |
|  | Nanjing | 66 | 4.68 | 0.469 | 0.058 | 4.57 | 4.80 | 4 | 5 |
|  | Shenyang | 66 | 4.27 | 0.795 | 0.098 | 4.08 | 4.47 | 2 | 5 |
|  | Xiamen | 66 | 3.91 | 0.799 | 0.098 | 3.71 | 4.11 | 2 | 5 |
|  | Total | 264 | 4.27 | 0.725 | 0.045 | 4.18 | 4.36 | 2 | 5 |
| Q7 (S2_12) | Xi'an | 66 | 4.21 | 0.448 | 0.055 | 4.10 | 4.32 | 3 | 5 |
|  | Nanjing | 66 | 4.47 | 0.503 | 0.062 | 4.35 | 4.59 | 4 | 5 |
|  | Shenyang | 66 | 4.17 | 0.954 | 0.117 | 3.93 | 4.40 | 2 | 5 |
|  | Xiamen | 66 | 4.05 | 0.753 | 0.093 | 3.86 | 4.23 | 2 | 5 |
|  | Total | 264 | 4.22 | 0.708 | 0.044 | 4.14 | 4.31 | 2 | 5 |
| Q8 (S2_13) | Xi'an | 66 | 4.26 | 0.474 | 0.058 | 4.14 | 4.37 | 3 | 5 |
|  | Nanjing | 66 | 4.29 | 0.548 | 0.067 | 4.15 | 4.42 | 3 | 5 |
|  | Shenyang | 66 | 4.21 | 0.851 | 0.105 | 4.00 | 4.42 | 2 | 5 |
|  | Xiamen | 66 | 3.48 | 0.846 | 0.104 | 3.28 | 3.69 | 2 | 5 |
|  | Total | 264 | 4.06 | 0.773 | 0.048 | 3.97 | 4.15 | 2 | 5 |
| Q9 (S2_16) | Xi'an | 66 | 4.09 | 0.650 | 0.080 | 3.93 | 4.25 | 3 | 5 |
|  | Nanjing | 66 | 4.36 | 0.671 | 0.083 | 4.20 | 4.53 | 2 | 5 |
|  | Shenyang | 66 | 4.03 | 0.859 | 0.106 | 3.82 | 4.24 | 2 | 5 |
|  | Xiamen | 66 | 3.20 | 0.827 | 0.102 | 2.99 | 3.40 | 2 | 5 |
|  | Total | 264 | 3.92 | 0.871 | 0.054 | 3.81 | 4.03 | 2 | 5 |
| Q10 (S2_17) | Xi'an | 66 | 4.03 | 0.607 | 0.075 | 3.88 | 4.18 | 3 | 5 |
|  | Nanjing | 66 | 4.02 | 0.620 | 0.076 | 3.86 | 4.17 | 3 | 5 |
|  | Shenyang | 66 | 4.24 | 0.842 | 0.104 | 4.04 | 4.45 | 2 | 5 |
|  | Xiamen | 66 | 2.23 | 1.161 | 0.143 | 1.94 | 2.51 | 1 | 5 |
|  | Total | 264 | 3.63 | 1.166 | 0.072 | 3.49 | 3.77 | 1 | 5 |

| **ANOVA** |  |  |  |  |  |  | **Effect size** |
| --- | --- | --- | --- | --- | --- | --- | --- |
|  |  | **Sum of Squares** | **df** | **Mean Square** | **F** | **Sig.** | **Eta Squared** |
| Number of expectation items | Between Groups | 54.83 | 3 | 18.277 | 44.759 | 0.000 | 0.341 |
|  | Within Groups | 106.167 | 260 | 0.408 |  |  |  |
|  | Total | 160.996 | 263 |  |  |  |  |
| Q1 (S2_5) | Between Groups | 107.405 | 3 | 35.802 | 48.747 | 0.000 | 0.360 |
|  | Within Groups | 190.955 | 260 | 0.734 |  |  |  |
|  | Total | 298.36 | 263 |  |  |  |  |
| Q2 (S2_6) | Between Groups | 22.864 | 3 | 7.621 | 13.096 | 0.000 | 0.131 |
|  | Within Groups | 151.303 | 260 | 0.582 |  |  |  |
|  | Total | 174.167 | 263 |  |  |  |  |
| Q3 (S2_7) | Between Groups | 39.227 | 3 | 13.076 | 16.98 | 0.000 | 0.164 |
|  | Within Groups | 200.212 | 260 | 0.77 |  |  |  |
|  | Total | 239.439 | 263 |  |  |  |  |
| Q4 (S2_9) | Between Groups | 20.318 | 3 | 6.773 | 13.523 | 0.000 | 0.135 |
|  | Within Groups | 130.212 | 260 | 0.501 |  |  |  |
|  | Total | 150.53 | 263 |  |  |  |  |
| Q5 (S2_10) | Between Groups | 36.648 | 3 | 12.216 | 23.155 | 0.000 | 0.211 |
|  | Within Groups | 137.167 | 260 | 0.528 |  |  |  |
|  | Total | 173.814 | 263 |  |  |  |  |
| Q6 (S2_11) | Between Groups | 19.909 | 3 | 6.636 | 14.566 | 0.000 | 0.144 |
|  | Within Groups | 118.455 | 260 | 0.456 |  |  |  |
|  | Total | 138.364 | 263 |  |  |  |  |
| Q7 (S2_12) | Between Groups | 6.314 | 3 | 2.105 | 4.361 | 0.005 | 0.048 |
|  | Within Groups | 125.5 | 260 | 0.483 |  |  |  |
|  | Total | 131.814 | 263 |  |  |  |  |
| Q8 (S2_13) | Between Groups | 29.364 | 3 | 9.788 | 19.934 | 0.000 | 0.187 |
|  | Within Groups | 127.667 | 260 | 0.491 |  |  |  |
|  | Total | 157.03 | 263 |  |  |  |  |
| Q9 (S2_16) | Between Groups | 50.223 | 3 | 16.741 | 29.192 | 0.000 | 0.252 |
|  | Within Groups | 149.106 | 260 | 0.573 |  |  |  |
|  | Total | 199.33 | 263 |  |  |  |  |
| Q10 (S2_17) | Between Groups | 174.985 | 3 | 58.328 | 83.036 | 0.000 | 0.489 |
|  | Within Groups | 182.636 | 260 | 0.702 |  |  |  |
|  | Total | 357.621 | 263 |  |  |  |  |

**B: Comparing the variances of acceptability of smart nursing homes in cities**

|  |  | **N** | **Mean** | **Std. Deviation** | **Std. Error** | **95% Confidence Interval for Mean** |  | **Minimum** | **Maximum** |
| --- | --- | --- | --- | --- | --- | --- | --- | --- | --- |
|  |  |  |  |  |  | Lower Bound | Upper Bound |  |  |
| Number of acceptability items | Xi'an | 66 | 2.03 | 0.701 | 0.086 | 1.86 | 2.20 | 1 | 3 |
|  | Nanjing | 66 | 2.47 | 0.588 | 0.072 | 2.33 | 2.61 | 1 | 3 |
|  | Shenyang | 66 | 2.12 | 0.903 | 0.111 | 1.90 | 2.34 | 1 | 3 |
|  | Xiamen | 66 | 1.21 | 0.448 | 0.055 | 1.10 | 1.32 | 1 | 3 |
|  | Total | 264 | 1.96 | 0.819 | 0.050 | 1.86 | 2.06 | 1 | 3 |
| Q11 (S3_1_1) | Xi'an | 66 | 3.36 | 0.853 | 0.105 | 3.15 | 3.57 | 1 | 5 |
|  | Nanjing | 66 | 3.50 | 0.685 | 0.084 | 3.33 | 3.67 | 2 | 5 |
|  | Shenyang | 66 | 3.76 | 0.658 | 0.081 | 3.60 | 3.92 | 2 | 5 |
|  | Xiamen | 66 | 2.62 | 1.049 | 0.129 | 2.36 | 2.88 | 1 | 5 |
|  | Total | 264 | 3.31 | 0.924 | 0.057 | 3.20 | 3.42 | 1 | 5 |
| Q12 (S3_1_2) | Xi'an | 66 | 3.98 | 0.668 | 0.082 | 3.82 | 4.15 | 3 | 5 |
|  | Nanjing | 66 | 4.14 | 0.630 | 0.078 | 3.98 | 4.29 | 3 | 5 |
|  | Shenyang | 66 | 4.02 | 0.411 | 0.051 | 3.91 | 4.12 | 3 | 5 |
|  | Xiamen | 66 | 2.80 | 0.964 | 0.119 | 2.57 | 3.04 | 1 | 5 |
|  | Total | 264 | 3.73 | 0.880 | 0.054 | 3.63 | 3.84 | 1 | 5 |
| Q13 (S3_1_3) | Xi'an | 66 | 4.45 | 0.637 | 0.078 | 4.30 | 4.61 | 3 | 5 |
|  | Nanjing | 66 | 4.59 | 0.526 | 0.065 | 4.46 | 4.72 | 3 | 5 |
|  | Shenyang | 66 | 4.42 | 0.609 | 0.075 | 4.27 | 4.57 | 3 | 5 |
|  | Xiamen | 66 | 3.23 | 0.908 | 0.112 | 3.00 | 3.45 | 1 | 5 |
|  | Total | 264 | 4.17 | 0.876 | 0.054 | 4.07 | 4.28 | 1 | 5 |
| Q14 (S3_1_4) | Xi'an | 66 | 4.45 | 0.637 | 0.078 | 4.30 | 4.61 | 2 | 5 |
|  | Nanjing | 66 | 4.20 | 0.661 | 0.081 | 4.03 | 4.36 | 3 | 5 |
|  | Shenyang | 66 | 4.26 | 0.771 | 0.095 | 4.07 | 4.45 | 2 | 5 |
|  | Xiamen | 66 | 3.09 | 0.940 | 0.116 | 2.86 | 3.32 | 1 | 5 |
|  | Total | 264 | 4.00 | 0.927 | 0.057 | 3.89 | 4.11 | 1 | 5 |
| Q15 (S3_2_2) | Xi'an | 66 | 4.26 | 0.563 | 0.069 | 4.12 | 4.40 | 3 | 5 |
|  | Nanjing | 66 | 4.73 | 0.449 | 0.055 | 4.62 | 4.84 | 4 | 5 |
|  | Shenyang | 66 | 4.21 | 0.734 | 0.090 | 4.03 | 4.39 | 3 | 5 |
|  | Xiamen | 66 | 3.77 | 1.310 | 0.161 | 3.45 | 4.09 | 1 | 5 |
|  | Total | 264 | 4.24 | 0.895 | 0.055 | 4.13 | 4.35 | 1 | 5 |
| Q16 (S3_3_1) | Xi'an | 66 | 4.20 | 0.588 | 0.072 | 4.05 | 4.34 | 3 | 5 |
|  | Nanjing | 66 | 4.42 | 0.498 | 0.061 | 4.30 | 4.55 | 4 | 5 |
|  | Shenyang | 66 | 3.89 | 0.963 | 0.119 | 3.66 | 4.13 | 2 | 5 |
|  | Xiamen | 66 | 3.56 | 0.682 | 0.084 | 3.39 | 3.73 | 2 | 5 |
|  | Total | 264 | 4.02 | 0.772 | 0.048 | 3.93 | 4.11 | 2 | 5 |
| Q17 (S3_3_2) | Xi'an | 66 | 4.21 | 0.569 | 0.070 | 4.07 | 4.35 | 3 | 5 |
|  | Nanjing | 66 | 4.24 | 0.528 | 0.065 | 4.11 | 4.37 | 3 | 5 |
|  | Shenyang | 66 | 4.12 | 0.886 | 0.109 | 3.90 | 4.34 | 2 | 5 |
|  | Xiamen | 66 | 3.62 | 0.989 | 0.122 | 3.38 | 3.86 | 1 | 5 |
|  | Total | 264 | 4.05 | 0.805 | 0.050 | 3.95 | 4.15 | 1 | 5 |
| Q18 (S3_4_1) | Xi'an | 66 | 4.06 | 0.298 | 0.037 | 3.99 | 4.13 | 3 | 5 |
|  | Nanjing | 66 | 4.14 | 0.426 | 0.052 | 4.03 | 4.24 | 3 | 5 |
|  | Shenyang | 66 | 4.08 | 0.810 | 0.100 | 3.88 | 4.27 | 2 | 5 |
|  | Xiamen | 66 | 3.52 | 0.808 | 0.100 | 3.32 | 3.71 | 1 | 5 |
|  | Total | 264 | 3.95 | 0.673 | 0.041 | 3.87 | 4.03 | 1 | 5 |
| Q19 (S3_4_2) | Xi'an | 66 | 4.18 | 0.783 | 0.096 | 3.99 | 4.37 | 3 | 5 |
|  | Nanjing | 66 | 4.41 | 0.526 | 0.065 | 4.28 | 4.54 | 3 | 5 |
|  | Shenyang | 66 | 4.08 | 0.882 | 0.109 | 3.86 | 4.29 | 2 | 5 |
|  | Xiamen | 66 | 3.71 | 0.924 | 0.114 | 3.48 | 3.94 | 1 | 5 |
|  | Total | 264 | 4.09 | 0.829 | 0.051 | 3.99 | 4.20 | 1 | 5 |
| Q20 (S3_4_3) | Xi'an | 66 | 4.17 | 0.736 | 0.091 | 3.99 | 4.35 | 3 | 5 |
|  | Nanjing | 66 | 4.48 | 0.533 | 0.066 | 4.35 | 4.62 | 3 | 5 |
|  | Shenyang | 66 | 4.12 | 0.869 | 0.107 | 3.91 | 4.33 | 2 | 5 |
|  | Xiamen | 66 | 3.42 | 0.912 | 0.112 | 3.20 | 3.65 | 2 | 5 |
|  | Total | 264 | 4.05 | 0.864 | 0.053 | 3.94 | 4.15 | 2 | 5 |
| Q21 (S3_4_4) | Xi'an | 66 | 4.06 | 0.605 | 0.074 | 3.91 | 4.21 | 3 | 5 |
|  | Nanjing | 66 | 4.38 | 0.489 | 0.060 | 4.26 | 4.50 | 4 | 5 |
|  | Shenyang | 66 | 4.20 | 0.948 | 0.117 | 3.96 | 4.43 | 2 | 5 |
|  | Xiamen | 66 | 3.33 | 1.028 | 0.127 | 3.08 | 3.59 | 1 | 5 |
|  | Total | 264 | 3.99 | 0.889 | 0.055 | 3.88 | 4.10 | 1 | 5 |
| Q22 (S3_4_5) | Xi'an | 66 | 4.14 | 0.630 | 0.078 | 3.98 | 4.29 | 3 | 5 |
|  | Nanjing | 66 | 4.45 | 0.532 | 0.065 | 4.32 | 4.59 | 3 | 5 |
|  | Shenyang | 66 | 4.15 | 0.808 | 0.100 | 3.95 | 4.35 | 2 | 5 |
|  | Xiamen | 66 | 3.61 | 0.959 | 0.118 | 3.37 | 3.84 | 1 | 5 |
|  | Total | 264 | 4.09 | 0.806 | 0.050 | 3.99 | 4.18 | 1 | 5 |
| Q23 (S3_4_6) | Xi'an | 66 | 4.33 | 0.616 | 0.076 | 4.18 | 4.48 | 3 | 5 |
|  | Nanjing | 66 | 4.45 | 0.532 | 0.065 | 4.32 | 4.59 | 3 | 5 |
|  | Shenyang | 66 | 4.09 | 0.872 | 0.107 | 3.88 | 4.31 | 2 | 5 |
|  | Xiamen | 66 | 3.38 | 1.120 | 0.138 | 3.10 | 3.65 | 1 | 5 |
|  | Total | 264 | 4.06 | 0.914 | 0.056 | 3.95 | 4.18 | 1 | 5 |
| Q24 (S3_4_7) | Xi'an | 66 | 4.27 | 0.621 | 0.076 | 4.12 | 4.43 | 3 | 5 |
|  | Nanjing | 66 | 4.67 | 0.506 | 0.062 | 4.54 | 4.79 | 3 | 5 |
|  | Shenyang | 66 | 4.11 | 0.897 | 0.110 | 3.89 | 4.33 | 2 | 5 |
|  | Xiamen | 66 | 3.70 | 1.189 | 0.146 | 3.40 | 3.99 | 1 | 5 |
|  | Total | 264 | 4.19 | 0.910 | 0.056 | 4.08 | 4.30 | 1 | 5 |

| **ANOVA** |  |  |  |  |  |  | **Effect size** |
| --- | --- | --- | --- | --- | --- | --- | --- |
|  |  | **Sum of Squares** | **df** | **Mean Square** | **F** | **Sig.** | **Eta Squared** |
| Number of acceptability items | Between Groups | 56.102 | 3 | 18.701 | 40.370 | 0.000 | 0.318 |
|  | Within Groups | 120.439 | 260 | 0.463 |  |  |  |
|  | Total | 176.542 | 263 |  |  |  |  |
| Q11 (S3_1_1) | Between Groups | 47.106 | 3 | 15.702 | 23.010 | 0.000 | 0.210 |
|  | Within Groups | 177.424 | 260 | 0.682 |  |  |  |
|  | Total | 224.530 | 263 |  |  |  |  |
| Q12 (S3_1_2) | Between Groups | 77.258 | 3 | 25.753 | 53.064 | 0.000 | 0.380 |
|  | Within Groups | 126.182 | 260 | 0.485 |  |  |  |
|  | Total | 203.439 | 263 |  |  |  |  |
| Q13 (S3_1_3) | Between Groups | 79.955 | 3 | 26.652 | 56.784 | 0.000 | 0.396 |
|  | Within Groups | 122.030 | 260 | 0.469 |  |  |  |
|  | Total | 201.985 | 263 |  |  |  |  |
| Q14 (S3_1_4) | Between Groups | 75.121 | 3 | 25.040 | 43.151 | 0.000 | 0.332 |
|  | Within Groups | 150.879 | 260 | 0.580 |  |  |  |
|  | Total | 226.000 | 263 |  |  |  |  |
| Q15 (S3_2_2) | Between Groups | 30.152 | 3 | 10.051 | 14.491 | 0.000 | 0.143 |
|  | Within Groups | 180.333 | 260 | 0.694 |  |  |  |
|  | Total | 210.485 | 263 |  |  |  |  |
| Q16 (S3_3_1) | Between Groups | 27.830 | 3 | 9.277 | 18.686 | 0.000 | 0.177 |
|  | Within Groups | 129.076 | 260 | 0.496 |  |  |  |
|  | Total | 156.905 | 263 |  |  |  |  |
| Q17 (S3_3_2) | Between Groups | 16.648 | 3 | 5.549 | 9.386 | 0.000 | 0.098 |
|  | Within Groups | 153.712 | 260 | 0.591 |  |  |  |
|  | Total | 170.360 | 263 |  |  |  |  |
| Q18 (S3_4_1) | Between Groups | 16.621 | 3 | 5.540 | 14.035 | 0.000 | 0.139 |
|  | Within Groups | 102.636 | 260 | 0.395 |  |  |  |
|  | Total | 119.258 | 263 |  |  |  |  |
| Q19 (S3_4_2) | Between Groups | 16.708 | 3 | 5.569 | 8.834 | 0.000 | 0.092 |
|  | Within Groups | 163.924 | 260 | 0.630 |  |  |  |
|  | Total | 180.633 | 263 |  |  |  |  |
| Q20 (S3_4_3) | Between Groups | 39.557 | 3 | 13.186 | 21.863 | 0.000 | 0.201 |
|  | Within Groups | 156.803 | 260 | 0.603 |  |  |  |
|  | Total | 196.360 | 263 |  |  |  |  |
| Q21 (S3_4_4) | Between Groups | 41.591 | 3 | 13.864 | 21.663 | 0.000 | 0.200 |
|  | Within Groups | 166.394 | 260 | 0.640 |  |  |  |
|  | Total | 207.985 | 263 |  |  |  |  |
| Q22 (S3_4_5) | Between Groups | 24.617 | 3 | 8.206 | 14.575 | 0.000 | 0.144 |
|  | Within Groups | 146.379 | 260 | 0.563 |  |  |  |
|  | Total | 170.996 | 263 |  |  |  |  |
| Q23 (S3_4_6) | Between Groups | 45.890 | 3 | 15.297 | 22.855 | 0.000 | 0.209 |
|  | Within Groups | 174.015 | 260 | 0.669 |  |  |  |
|  | Total | 219.905 | 263 |  |  |  |  |
| Q24 (S3_4_7) | Between Groups | 31.951 | 3 | 10.650 | 14.891 | 0.000 | 0.147 |
|  | Within Groups | 185.955 | 260 | 0.715 |  |  |  |
|  | Total | 217.905 | 263 |  |  |  |  |

^1^ The item number on the final questionnaire (24 items)

^2^ The item number on the second version of the questionnaire (40 items)
